# Copper catalyzed synthesis of thiazole derivatives from enaminones, amines and CS₂

**DOI:** 10.1038/s41598-026-40393-x

**Published:** 2026-02-16

**Authors:** Amin Arman, Najmeh Nowrouzi, Mohammad Abbasi

**Affiliations:** Department of Chemistry, Faculty of Nano and Bio Science and Technology, Persian Gulf University, Bushehr, 75169 Iran

**Keywords:** C–H activation, Thiazoles, Copper catalysts, Enaminone, Amine, Chemical biology, Chemistry

## Abstract

An efficient copper-catalyzed method for the synthesis of thiazoles from enaminones, amines, and carbon disulfide (CS₂) is described. This one-pot, ligand- and additive-free protocol proceeds under relatively simple reaction conditions, affording thiazole derivatives in good to excellent yields. Notably, CS₂ serves as a combined sulfur and carbon source, contributing to the synthetic practicality of the transformation. The method demonstrates good functional-group tolerance and provides a straightforward approach to biologically relevant thiazole scaffolds.

**Supplementary Information:**

The online version contains supplementary material available at 10.1038/s41598-026-40393-x.

## Introduction

The incorporation of both nitrogen and sulfur atoms into molecular frameworks often imparts unique chemical properties and diverse biological activities. Such nitrogen–sulfur heterocycles have attracted significant attention due to their versatile reactivity and their potential applications in pharmaceuticals, catalysis, and materials science^1^.Among these heterocycles, thiazole—a five-membered ring containing both sulfur and nitrogen—has emerged as a privileged scaffold, combining distinctive electronic properties with remarkable structural adaptability.

Thiazole derivatives exhibit a wide spectrum of bioactivities, including antimicrobial (antibacterial, antifungal, antimalarial), anti-inflammatory, analgesic, anticancer, cardiovascular, neurological, and antioxidant effects. This diverse pharmacological profile arises from the unique electronic configuration of the thiazole nucleus and its ability to mimic essential biological motifs in drug–target interactions^2–4^. In particular, 2-aminobenzothiazoles are important benzothiazole derivatives extensively studied in bioorganic and medicinal chemistry, finding applications in drug discovery and development for diseases such as AIDS, diabetes, epilepsy, and tuberculosis^5^. Furthermore, *N*-arylbenzo[d]thiazol-2-amines constitute key motifs in anticancer and antimicrobial agents^6^.

Given their remarkable properties, considerable efforts have been directed toward developing environmentally friendly and operationally simple synthetic strategies for thiazoles. Recent advances, including metal-free cyclization^7^ and photocatalytic C–H sulfuration^8^, have broadened access to novel thiazole frameworks with potential applications in OLEDs, conductive polymers, and organocatalysis^9–11^. Traditionally, thiazole and benzothiazole derivatives have been prepared through the condensation of 2-aminothiophenols with aryl ketones^12^, nitriles^13^, benzylamines^14^, benzyl chlorides^15^, or *β*-ketoesters^16^. Additionally, transition-metal-catalyzed C–H activation and subsequent functionalization with aryl halides have become well-established methods for constructing benzothiazole cores^17–19^.

Several practical protocols have also been developed to improve efficiency and selectivity. For example, Ding and co-workers reported a copper-catalyzed synthesis of 2-aminobenzothiazoles from 2-halobenzenamines and isothiocyanates^20^. The Wang group described the preparation of 2-aminophenyl benzothiazoles *via* the reaction of 2-aminobenzenethiol with isothiocyanates in the presence of Fe(NO_3_)_3_·9H_2_O^21^. More recently, Murthy Boddapati and colleagues introduced a convenient copper-catalyzed route using aryl isothioureas and aryl iodides^22^, while Zhang et al.. employed 2-iodoanilines and styrene, with elemental sulfur acting as both an oxidant and a one-carbon donor^23^. Despite these advances, most methods focus on benzothiazoles, and only a few protocols have been reported for the synthesis of thiazoles from enaminones^24–27^. Therefore, developing a straightforward and efficient strategy for constructing thiazoles based on enaminones remains highly desirable, offering a promising avenue for accessing structurally diverse thiazole derivatives with potential applications in medicinal chemistry and materials science.

**Scheme 1:**
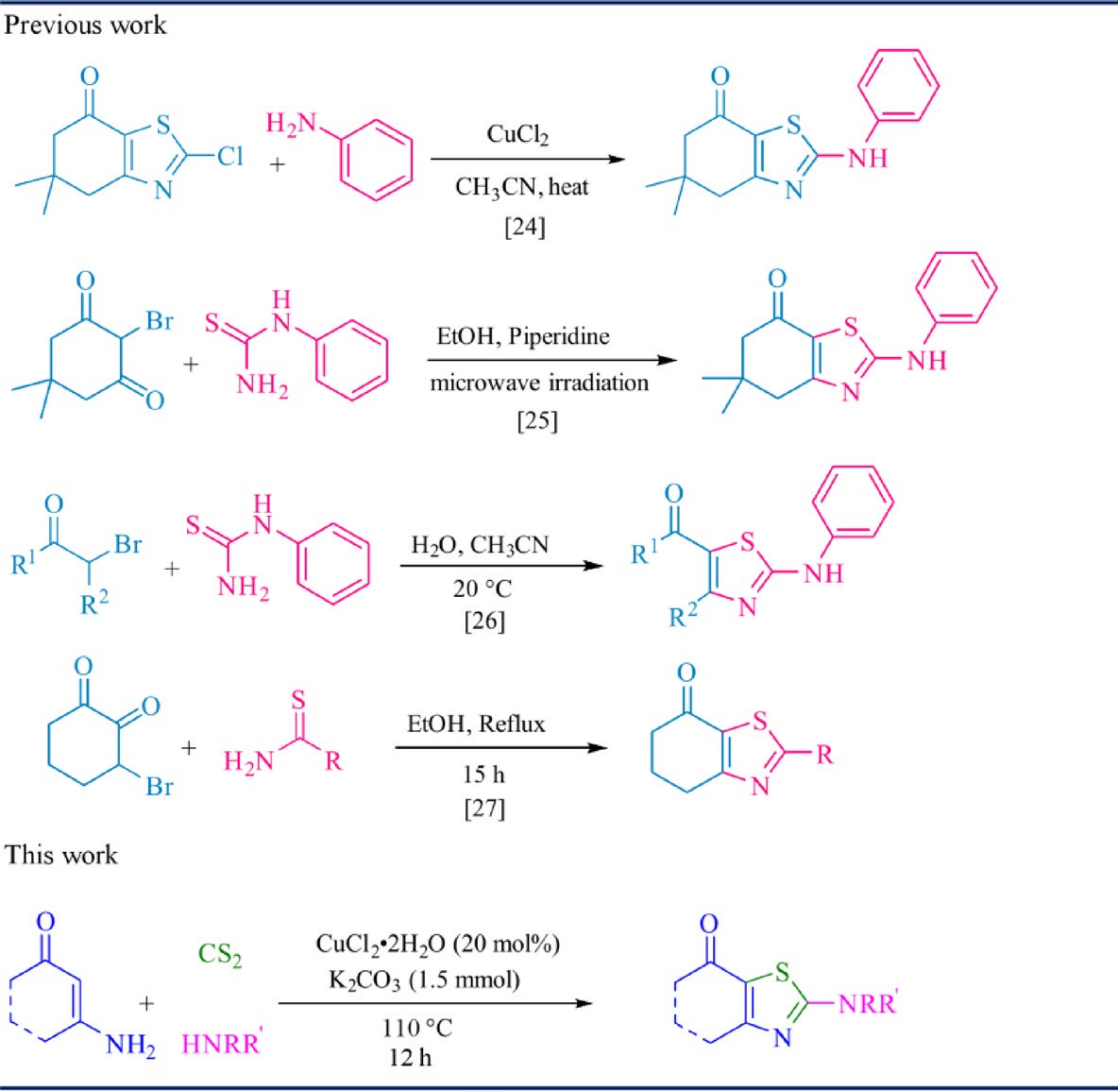
Pathways for the synthesis of thiazoles.

Building on these advances, we have developed a direct and efficient method for the construction of thiazoles *via* simultaneous C–S and C–N bond formation from enaminones, amines, and CS_2_ under copper-catalyzed conditions (Scheme 1). Notably, this straightforward oxidative coupling and cyclization proceeds smoothly without the need for any additional ligands, additives, or external oxidants, highlighting its operational simplicity and practical applicability.

## Results and discussion

Our initial studies began with the addition of CS_2_ (0.75 mmol) to a mixture of *N*-methyl-1-phenylmethanamine (0.75 mmol), and K_2_CO_3_ as the base (1.0 mmol) in DMSO (1 mL) as the solvent. The mixture was kept under stirring at ambient temperature for 30 min. Then, enaminone (0.5 mmol), and CuCl₂·2 H₂O (0.1 mmol) were subsequently added to the reaction mixture, which was stirred at 100 °C for 12 h, affording the desired product in a moderate 65% yield (Table 1, entry 1). Switching the solvent to dimethylformamide (DMF) improved the yield to 76% under identical conditions (Table 1, entry 2), while alternative solvents such as 1,4-dioxane, ethanol, water, PEG-200, toluene, and acetonitrile led to only trace or low product formation (Table 1, entries 3–7). Temperature screening revealed that raising the reaction temperature to 110 °C resulted in a slight improvement in yield (Table 1, entry 8), whereas further raising it to 120 °C did not provide any significant benefit (Table 1, entry 9). Among the bases tested, K₂CO₃ proved to be the most effective (Table 1, entries 10–15), and increasing its amount from 1.0 mmol to 1.5 mmol further enhanced the yield to 90% (Table 1, entry 16). Additional increments in base loading did not significantly affect the outcome (Table 1, entry 17), whereas reducing the base to 0.5 mmol resulted in decreased conversion (Table 1, entry 18). As expected, no significant product formation was observed in the absence of a base (Table 1, entry 19). Furthermore, a range of transition-metal catalysts, including CuI, CuCl, Cu(OAc)₂, CoCl_2_, NiCl₂·6 H₂O, and MnCl₂, were evaluated, and CuCl₂·2 H₂O was identified as the most efficient, delivering the product in 90% yield (Table 1, entries 20–25). Reducing the catalyst loading led to a decline in yield, highlighting the importance of optimized conditions (Table 1, entry 26). On the other hand, increasing the amount of CuCl₂·2 H₂O up to 30 mol% did not have a significant enhancement on the yield (Table 1, entry 27).

Therefore, the optimal conditions for the synthesis of thiazoles were established as CuCl₂·2 H₂O (20 mol%) as the catalyst, K₂CO₃ (1.5 mmol) as the base, DMF as the solvent, and the reaction carried out at 110 °C for 12 h. Following the successful preparation of the desired product **4a**, we turned our attention to the synthesis of a series of thiazole derivatives under these optimized conditions (Table 1, entry 16).

**Table 1:** Optimization the reaction conditions^a^.


Entry	Catalyst [mol%]	Solvent	Base [mmol]	Temp [˚C]	Yield [%]^a^
1	CuCl_2_·2H_2_O (20 mol%)	DMSO	K_2_CO_3_ (1.0 mmol)	100	65
2	CuCl_2_·2H_2_O (20 mol%)	DMF	K_2_CO_3_ (1.0 mmol)	100	76
3	CuCl_2_·2H_2_O (20 mol%)	1,4-dioxane	K_2_CO_3_ (1.0 mmol)	100	22
4	CuCl_2_·2H_2_O (20 mol%)	EtOH	K_2_CO_3_ (1.0 mmol)	100	Trace
5	CuCl_2_·2H_2_O (20 mol%)	H_2_O	K_2_CO_3_ (1.0 mmol)	100	Trace
6	CuCl_2_·2H_2_O (20 mol%)	PEG-200	K_2_CO_3_ (1.0 mmol)	100	Trace
6	CuCl_2_·2H_2_O (20 mol%)	Toluene	K_2_CO_3_ (1.0 mmol)	100	36
7	CuCl_2_·2H_2_O (20 mol%)	CH_3_CN	K_2_CO_3_ (1.0 mmol)	100	15
8	CuCl_2_·2H_2_O (20 mol%)	DMF	K_2_CO_3_ (1.0 mmol)	110	81
9	CuCl_2_·2H_2_O (20 mol%)	DMF	K_2_CO_3_ (1.0 mmol)	120	81
10	CuCl_2_·2H_2_O (20 mol%)	DMF	Na_2_CO_3_ (1.0 mmol)	110	77
11	CuCl_2_·2H_2_O (20 mol%)	DMF	NaHCO_3_ (1.0 mmol)	120	70
12	CuCl_2_·2H_2_O (20 mol%)	DMF	NaOH (1.0 mmol)	110	61
13	CuCl_2_·2H_2_O (20 mol%)	DMF	KOH (1.0 mmol)	110	75
14	CuCl_2_·2H_2_O (20 mol%)	DMF	Et_3_N (1.0 mmol)	110	43
15	CuCl_2_·2H_2_O (20 mol%)	DMF	‌DABCO (1.0 mmol)	110	38
**16**	**CuCl**_**2**_ **·2 H**_**2**_ **O (20 mol%)**	**DMF**	**K**_**2**_**CO**_**3**_ **(1.5 mmol)**	**110**	**90**
17	CuCl_2_·2H_2_O (20 mol%)	DMF	K_2_CO_3_ (2.0 mmol)	110	90
18	CuCl_2_·2H_2_O (20 mol%)	DMF	K_2_CO_3_ (0.5 mmol)	110	54
19	CuCl_2_·2H_2_O (20 mol%)	DMF	-	110	Trace
20	CuI (20 mol%)	DMF	K_2_CO_3_ (1.5 mmol)	110	49
21	CuCl (20 mol%)	DMF	K_2_CO_3_ (1.5 mmol)	110	52
22	Cu(OAc)_2_ (20 mol%)	DMF	K_2_CO_3_ (1.5 mmol)	110	37
23	CoCl_2_ (20 mol%)	DMF	K_2_CO_3_ (1.5 mmol)	110	Trace
24	NiCl_2_·6H_2_O (20 mol %)	DMF	K_2_CO_3_ (1.5 mmol)	110	Trace
25	‌ MnCl_2_ (20 mol%)	DMF	K_2_CO_3_ (1.5 mmol)	110	19
26	CuCl_2_·2H_2_O (10 mol%)	DMF	K_2_CO_3_ (1.5 mmol)	110	47
27	CuCl_2_·2H_2_O (30 mol%)	DMF	K_2_CO_3_ (1.5 mmol)	110	90

^a^ Reaction conditions: First step: CS_2_ (0.75 mmol), *N*-methyl-1-phenylmethanamine (0.75 mmol), K_2_CO_3_ (1.5 mmol), Solvent (1 mL) at room temperature for 30 min; Second step: Enaminone (0.5 mmol), CuCl_2_·2H_2_O (20 mol%) at 110 °C for 12 h.

As illustrated in Table 2, we initially investigated the reaction of various secondary amines, such as *N*-methyl-1-phenylmethanamine, *N*-methylaniline, dibenzylamine, piperidine, pyrrolidine, diethylamine, dimethylamine, *N*-methylcyclohexanamine, and morpholine, with CS_2_ and enaminone. These reactions furnished the desired products **4a-4i** in good to excellent yields. Subsequently, aniline and its derivatives, including 3,4-dimethylaniline, *o*-toluidine, 4-chloroaniline, and 4-bromoaniline, were also reacted under the same conditions to provide products **4j-4n** in moderate yields. The results indicate that both the electronic nature and the position of substituents on the aniline rings have only a limited effect on this oxidative-coupling cyclization reaction. The successful synthesis of **4 L** from *o*-toluidine demonstrates that arylamines bearing ortho-substituents indicate acceptable reactivity, proposing that steric factors on the aryl ring have minimal influence on the reaction outcome.

When butan-1-amine and phenylmethanamine were employed under the optimized conditions, the expected products **4o** and **4p** were obtained in acceptable yields. To further explore the substrate scope, 3-aminocyclohex-2-en-1-one was treated with both secondary and primary amines, affording products **4q-4t** in good yields. A comparison between primary and secondary amines revealed that secondary aliphatic and aromatic amines generally exhibit higher reactivity, delivering the desired compounds in good to excellent yields, whereas primary aliphatic and aromatic amines provided slightly lower yields (Table 2).

Unfortunately, when 4-nitroaniline, pyridin-4-amine, and pyridin-2-amine were used as substrates, no corresponding products **4u–4w** were observed. Further attempts to promote these reactions in DMSO in the presence of a strong base such as KOH also failed. This lack of reactivity can be attributed to strong electronic deactivation in the case of 4-nitroaniline and pyridine amines, which significantly reduces the electron density on the aniline nitrogen through both inductive and resonance effects. This electronic deficiency adversely affects the reactivity of the sulfur-containing species generated in situ, leading to a substantial reduction in its nucleophilicity. As a consequence, nucleophilic attack on the electrophilic reaction partner becomes unfavorable, and the reaction does not proceed further (Scheme 3). The reaction of (E)−4-aminopent-3-en-2-one, a linear enaminone, with aniline and CS_2_ under the optimized conditions was investigated; however, the desired product **4x** was not formed. Probably, the low reactivity of linear enaminones originates from steric hindrance caused by their flexible conformations, which interferes with metal coordination and cyclization. To explore the scope of the reaction, the enaminone 6-amino-1,3-dimethylpyrimidine-2,4(1 H,3 H)-dione was reacted with *N*-methyl-1-phenylmethanamine and *N*-methylaniline, while 4-amino-2 H-chromen-2-one was treated with propan-1-amine under the optimized conditions. In all cases, the reactions proceeded efficiently and afforded the desired products (**4y**, **4z** and **4aa**) in excellent yields. These results indicate that the method is compatible with both aromatic and aliphatic amines, as well as with different enaminone frameworks.

Additional experimental details and characterization data are provided in Supplementary Table 2.

**Table 2:** Synthesis of thiazole derivativesa.


			‌
			
			
			
			
			
			

^a^Reaction conditions: First step: CS_2_ (0.75 mmol), Amines (0.75 mmol), K_2_CO_3_ (1.5 mmol), DMF (1 mL) at room

temperature for 30 min; Second step: Enaminones (0.5 mmol), CuCl_2_·2H_2_O (20 mol%), at 110 °C for 12 h.

**Scheme 2:**
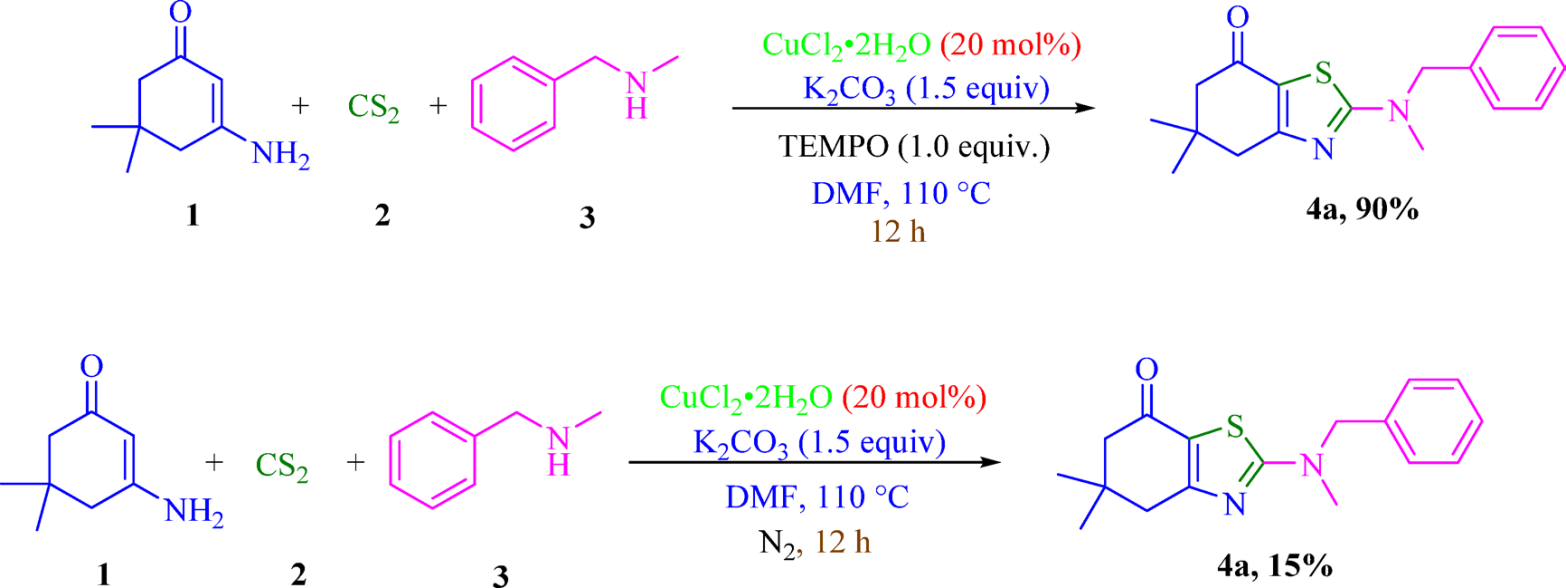
Control experiments.

To gain further insight into the reaction pathway and to clarify the possible involvement of radical species, a series of control experiments were conducted under the optimized reaction conditions (Scheme 2).

First, the model reaction was performed in the presence of 2,2,6,6-tetramethylpiperidine-1-oxyl (TEMPO) as a well-known radical scavenger. Notably, the reaction proceeded smoothly, affording the desired thiazole product in comparable yield and within a similar reaction time to that observed under standard conditions. The negligible effect of TEMPO on both reaction efficiency and rate strongly suggests that a radical pathway is unlikely to be operative in this transformation.

In contrast, when the reaction was carried out under an inert nitrogen atmosphere, a significant decrease in product yield was observed. This result indicates that molecular oxygen plays a crucial role in the reaction process. Considering the reduced efficiency under oxygen-free conditions, it is reasonable to propose that oxygen is involved in the regeneration of the catalytically active copper species, most likely through the reoxidation of Cu(I) to Cu(II).

**Scheme 3:**
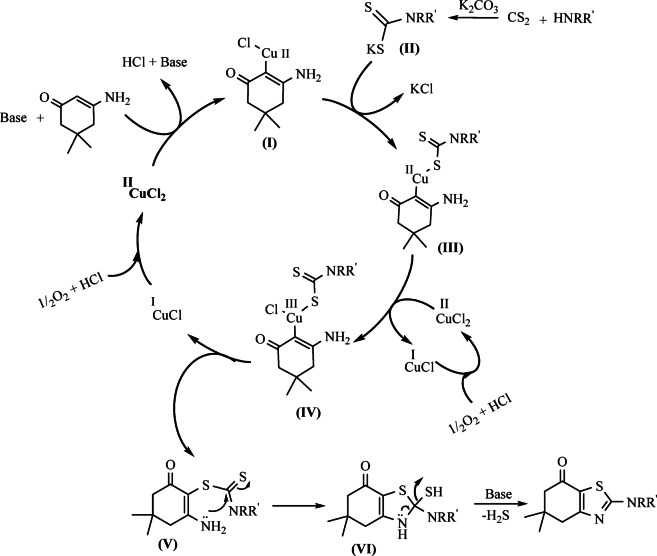
Proposed mechanism for the synthesis of thiazoles.

Based on the obtained results, control experiments, and previous literature reports on copper-catalyzed transformations of enaminones^28–33^, a plausible mechanism for the synthesis of thiazoles from enaminones, amines, and CS_2_ is proposed, as illustrated in Scheme 3.

The catalytic cycle is initiated by coordination of the enaminone to CuCl₂·2 H₂O through its *α*-carbon, forming activated complex **I**^28–33^, while the amine reacts with CS_2_ under basic conditions to produce the corresponding dithiocarbamate salt **II**^34–38^. Nucleophilic substitution of complex I with dithiocarbamate **II** affords intermediate **III**, which undergoes disproportionation of Cu(II) to Cu(III), generating species **IV**. Subsequent reductive elimination from **IV** provides intermediate **V** along with Cu(I). In **V**, the amino group intramolecularly attacks the electrophilic C = S unit to form the cyclized intermediate **VI**, which upon base-assisted elimination of hydrogen sulfide delivers the desired thiazole product. Finally, Cu(I) is reoxidized to Cu(II) by molecular oxygen in the presence of HCl, thereby regenerating the active catalyst and completing the catalytic cycle.

## Conclusion

In conclusion, a practical copper-catalyzed strategy for the synthesis of thiazoles from enaminones, amines, and CS₂ has been developed. The protocol operates under ligand- and additive-free conditions and shows good efficiency, particularly for secondary amines. this method provides a reliable and straightforward approach for accessing structurally diverse thiazole derivatives from readily available starting materials. This study expands the synthetic utility of enaminones in heterocycle construction.

## Exprimental

All chemicals needed in this study were supplied by Merck or Fluka chemical companies. ^1^H-NMR and^13^C-NMR spectra were run on a Bruker Avance 400 MHz instrument in CDCl_3_. Melting points were measured as a Buchi B-545 apparatus in open capillary tubes.

### Typical procedure for the synthesis of thiazoles

At first, a mixture of amine (0.75 mmol), CS_2_ (0.75 mmol), K₂CO₃ (1.5 mmol), and *N*, *N*-dimethylformamide (1 mL) was stirred at ambient temperature for 30 min. Next, after the addition of enaminone (0.5 mmol) and CuCl₂·2 H₂O (20 mol%), the resulting mixture was allowed to stir at 110 °C for 12 h. When the reaction was completed (monitored by TLC), the temperature was gradually decreased to room temperature, and the product was extracted with EtOAc (3 × 3 mL). After that, the solvent was eliminated under reduced pressure. Finally, the column chromatography was employed to purify the crude product using *n*-hexane/ethyl acetate (10:3) as the solvent to provide the desired product (Table 2).

## Supplementary Information

Below is the link to the electronic supplementary material.

Supplementary Material 1

## Data Availability

The data supporting this article have been included as part of the Supplementary Information.

## References

[1] Petkowski, J. J., Bains, W. & Seager, S. Natural products containing a nitrogen–sulfur bond. *J. Nat. Prod.* **81**, 423–446 (2018). 29364663 10.1021/acs.jnatprod.7b00921

[2] Petrou, A., Fesatidou, M. & Geronikaki, A. Thiazole ring—A biologically active scaffold. *Molecules* **26**, 3166 (2021). 34070661 10.3390/molecules26113166 PMC8198555

[3] Popsavin, M. et al. Synthesis and antiproliferative activity of two new Tiazofurin analogues with 2′-amido functionalities. *Bioorg. Med. Chem. Lett.* **16**, 2773–2776 (2006). 16495053 10.1016/j.bmcl.2006.02.001

[4] Sevrioukova, I. F. & Poulos, T. L. Dissecting cytochrome P450 3A4–ligand interactions using Ritonavir analogues. *Biochemistry* **52**, 4474–4481 (2013). 23746300 10.1021/bi4005396

[5] Zeng, W., Dang, P., Zhang, X., Liang, Y. & Peng, C. Copper-catalyzed synthesis of 2-aminobenzothiazoles from carbodiimide and sodium hydrosulfide. *RSC Adv.* **4**, 31003–31006 (2014).

[6] Thi, S. N. et al. Efficient synthesis of N-arylbenzo [d] thiazol-2-amine derivatives from benzo [d] thiazole-2-thiols under metal-free condition. *Tetrahedron Lett.* **126**, 154662 (2023).

[7] Xie, Y. et al. Metal-free oxidative cyclization of 2-aminobenzothiazoles and Cyclic ketones enabled by the combination of elemental sulfur and oxygen. *Green Chem.* **19**, 4294–4298 (2017).

[8] Dinh, A. N. et al. Photocatalytic oxidative C–H thiolation: synthesis of benzothiazoles and sulfenylated Indoles. *Synlett* **30**, 1648–1655 (2019).

[9] Lin, Y., Fan, H., Li, Y. & Zhan, X. Thiazole-based organic semiconductors for organic electronics. *Adv. Mater.* **24**, 3087–3106 (2012). 22581766 10.1002/adma.201200721

[10] Yuan, Z. et al. Unipolar electron transport polymers: a thiazole based all-electron acceptor approach. *Chem. Mater.* **28**, 6045–6049 (2016).

[11] Hou, J. & Kazemi, M. A comprehensive review on synthesis of oxazoles: research on magnetically recoverable catalysts. *Research Chem. Intermediates*. **50**, 1845–1872 (2024).

[12] Liao, Y. et al. Efficient 2-aryl benzothiazole formation from Aryl ketones and 2-aminobenzenethiols under metal-free conditions. *Org. Lett.* **14**, 6004–6007 (2012). 23151061 10.1021/ol302902e

[13] Sun, Y., Jiang, H., Wu, W., Zeng, W. & Wu, X. Copper-catalyzed synthesis of substituted benzothiazoles via condensation of 2-aminobenzenethiols with nitriles. *Org. Lett.* **15**, 1598–1601 (2013). 23496117 10.1021/ol400379z

[14] Xiao, T., Xiong, S., Xie, Y., Dong, X. & Zhou, L. Copper-catalyzed synthesis of Benzazoles via aerobic oxidative condensation of o-amino/mercaptan/hydroxyanilines with benzylamines. *RSC Adv.* **3**, 15592–15595 (2013).

[15] Gan, H. et al. S8-Mediated cyclization of 2‐Aminophenols/thiophenols with arylmethyl chloride: approach to Benzoxazoles and benzothiazoles. *Chemistry–An Asian J.* **11**, 1770–1774 (2016). 10.1002/asia.201600355 27124616

[16] Li, Z. et al. Metal-and oxidant-free synthesis of Quinazolinones from β-ketoesters with o-aminobenzamides via phosphorous acid-catalyzed cyclocondensation and selective C–C bond cleavage. *J. Org. Chem.* **80**, 9392–9400 (2015). 26339716 10.1021/acs.joc.5b00937

[17] Gao, Y., Song, Q., Cheng, G. & Cui, X. KI-catalyzed Arylation of benzothiazoles from the coupling of Aryl aldehydes with benzothiazoles in neat water. *Org. Biomol. Chem.* **12**, 1044–1047 (2014). 24407277 10.1039/c3ob42318b

[18] Dai, W. C. & Wang, Z. X. Palladium-catalyzed coupling of Azoles with 1-aryltriazenes via C–H/C–N cleavage. *Org. Chem. Front.* **4**, 1281–1288 (2017).

[19] Zhou, P. X. et al. Palladium/copper-catalyzed decarbonylative heteroarylation of amides via C–N bond activation. *Org. Chem. Front.* **6**, 1942–1947 (2019).

[20] Ding, Q., He, X. & Wu, J. Synthesis of 2-aminobenzothiazole via copper (I)-catalyzed tandem reaction of 2-iodobenzenamine with isothiocyanate. *J. Comb. Chem.* **11**, 587–591 (2009). 19449803 10.1021/cc900027c

[21] Wang, W. et al. Iron-catalyzed one-pot synthesis of 2-aminobenzothiazoles from 2-aminobenzethiols and isothiocyanates under ligand-free conditions in water. *Heterocycles* **81**, 2841–2847 (2010).

[22] MurthyáBoddapati, S., MohanáKurmarayuni, C., RamanaáMutchu, B. & BabuáBollikolla, H. Copper-catalyzed synthesis of 2-aminophenyl benzothiazoles: A novel approach. *Org. Biomol. Chem.* **16**, 8267–8272 (2018). 30215652 10.1039/c8ob02018c

[23] Zhang, J. et al. Elemental sulfur-promoted benzoxazole/benzothiazole formation using a C C double bond as a one-carbon donator. *J. Org. Chem.* **86**, 14485–14492 (2021). 34661400 10.1021/acs.joc.1c01357

[24] Stepanov, D., Ivanov, E. & Ivanova, R. Y. New derivatives of 4, 5, 6, 7-tetrahydrobenzothiazol-7-one and 5, 6, 7, 8-tetrahydro-4H-thiazolo [5, 4-c] azepin-8-one. *Russ. J. Gen. Chem.* **70**, 784–787 (2000).

[25] Dabholkar, V. V. & Mishra, S. K. J. Microwave-mediated synthesis of some novel heterocycles containing thiazole, Oxazole thiazine, oxazine, Thiadiazine and triazolo-thiadiazine moiety. *INDIAN J. Chem. Sect. B*. **45**, 2112 (2006).

[26] Yella, R., Kavala, V. & Patel, B. K. Bromineless bromine as an efficient desulfurizing agent for the Preparation of cyanamides and 2-aminothiazoles from dithiocarbamate salts. *Synth. Commun.* **41**, 792–805 (2011).

[27] Guernon, J. M. & Wu, Y. J. 3-Bromocyclohexane-1, 2-dione as a useful reagent for Hantzsch synthesis of thiazoles and the synthesis of related heterocycles. *Tetrahedron Lett.* **52**, 3633–3635 (2011).

[28] Bernini, R., Cacchi, S., Fabrizi, G., Filisti, E. & Sferrazza, A. 3-Aroylindoles via Copper-Catalyzed Cyclization of N-(2-Iodoaryl) enaminones. Synlett. **2009**, 1480–1484 (2009).

[29] Bernini, R., Fabrizi, G., Sferrazza, A., Cacchi, S. & Copper-Catalyzed, C-C. Bond formation through C-H functionalization: synthesis of multisubstituted Indoles from N‐Aryl enaminones. *Angew. Chem. Int. Ed.* **48**, 8078–8081 (2009). 10.1002/anie.200902440 19774575

[30] Fu, L., Liu, Y. & Wan, J. P. Pd-catalyzed triple-fold C (sp2)–H activation with enaminones and alkenes for pyrrole synthesis via hydrogen evolution. *Org. Lett.* **23**, 4363–4367 (2021). 34013729 10.1021/acs.orglett.1c01301

[31] Bashkar, M., Nowrouzi, N. & Mohammadizadeh, M. R. Copper-catalyzed intramolecular annulation through C–H activation: synthesis of carbazolones. *Appl. Organomet. Chem.* **37**, e7105 (2023).

[32] Fu, L., Wan, J. P., Zhou, L. & Liu, Y. Copper-catalyzed C–H/N–H annulation of enaminones and alkynyl esters for densely substituted pyrrole synthesis. *Chem. Commun.* **58**, 1808–1811 (2022). 10.1039/d1cc06768k 35040446

[33] Fu, L. et al. Copper (II)-Catalyzed [2 + 2+ 2] annulation of enaminones with maleimides using a traceless directing group strategy. *Adv. Synth. Catal.* **366**, 4139–4144 (2024).

[34] Monge, A. et al. Synthesis of 2-piperazinylbenzothiazole and 2-piperazinylbenzoxazole derivatives with 5-HT3 antagonist and 5-HT4 agonist properties. *J. Med. Chem.* **37**, 1320–1325 (1994). 8176710 10.1021/jm00035a012

[35] Li, G. et al. Photocatalyst-free visible-light-promoted C (sp2)–S coupling: a strategy for the Preparation of S-aryl dithiocarbamates. *Org. Lett.* **21**, 7938–7942 (2019). 31553199 10.1021/acs.orglett.9b02921

[36] Vishwakarma, R. K., Kumar, S. & Singh, K. N. Visible-light-induced photocatalytic synthesis of β-keto dithiocarbamates via difunctionalization of styrenes. *Org. Lett.* **23**, 4147–4151 (2021). 33988029 10.1021/acs.orglett.1c01059

[37] Li, X. et al. Three-component reaction access to S-alkyl dithiocarbamates under visible-light irradiation conditions in water. *Green Chem.* **24**, 1302–1307 (2022).

[38] Xu, H. et al. An electron donor–acceptor photoactivation strategy for the synthesis of S-aryl dithiocarbamates using thianthrenium salts under mild aqueous micellar conditions. *Chin. Chem. Lett.* **34**, 108403 (2023).

