# Supplementary material for: Copper catalyzed synthesis of thiazole derivatives from enaminones, amines and CS₂
Source: Sci Rep. 2026 Feb 16;16:9184. doi: 10.1038/s41598-026-40393-x (PMC12996597; doi:10.1038/s41598-026-40393-x)
Supplement: Supplementary file 1 — Supplementary Material 1 [file 41598_2026_40393_MOESM1_ESM.docx]

Supporting Information for

**Copper Catalyzed Synthesis of Thiazole Derivatives from Enaminones, Amines and CS₂**

Amin Arman,^a^ Najmeh Nowrouzi,*^a^ Mohammad Abbasi^a^

^a^ Department of Chemistry, Faculty of Nano and Bio Science and Technology, Persian Gulf University, Bushehr 75169 Iran

Email: nowrouzi@pgu.ac.ir

**2-(benzyl(methyl)amino)-5,5-dimethyl-5,6-dihydrobenzo[*d*]thiazol-7(4H)-one (Table 2, 4a).**

Light yellow powder; 90% yield; m.p. = 138-140 ^o^C; ^1^H-NMR (400 MHz, CDCl_3_) δ (ppm): 1.15 (s, 6H, CH_3_), 2.41 (s, 2H, CH_2_), 2.73 (s, 2H, CH_2_), 3.12 (s, 3H, CH_3_), 4.79 (s, 2H, CH_2_), 7.27-7.39 (m, 5H, Ar). ^13^C-NMR (100 MHz, CDCl_3_) δ (ppm): 28.6, 34.8, 38.2, 41.6, 51.3, 56.5, 119.0, 127.7, 128.0, 128.9, 135.5, 167.0, 175.3, 190.2. MS (*m/z*): 300 [M^+^], 271, 209, 120, 91, 65.

**5,5-dimethyl-2-(methyl(phenyl)amino)-5,6-dihydrobenzo[*d*]thiazol-7(4H)-one (Table 2, 4b).**

Cream powder; 88% yield; m.p. = 122-124 ^o^C; ^1^H-NMR (400 MHz, CDCl_3_) δ (ppm): 1.15 (s, 6H, CH_3_), 2.38 (s, 2H, CH_2_), 2.75 (s, 2H, CH_2_), 3.61 (s, 3H, CH_3_), 7.35-7.50 (m, 5H, Ar). ^13^C-NMR (100 MHz, CDCl_3_) δ (ppm): 28.6, 34.8, 40.7, 41.5, 51.3, 119.6, 125.6, 128.0, 130.2, 145.1, 166.3, 174.9, 190.6. Anal. Calcd for C₁₆H₁₈N₂OS: C, 67.10; H, 6.34; N, 9.78; O, 5.59; S, 11.19. Found: C, 67.01; H, 6.39; N, 9.88; S, 11.12.

**2-(dibenzylamino)-5,5-dimethyl-5,6-dihydrobenzo[*d*]thiazol-7(4H)-one (Table 2, 4c).**

Cream powder; 88% yield; m.p. = 108-111 ^o^C; ^1^H-NMR (400 MHz, CDCl_3_) δ (ppm): 1.17 (s, 6H, CH_3_), 2.42 (s, 2H, CH_2_), 2.75 (s, 2H, CH_2_), 4.72 (s, 4H, CH_2_), 7.24-7.40 (m, 10H, Ar). ^13^C-NMR (100 MHz, CDCl_3_) δ (ppm): 28.6, 34.8, 41.6, 51.3, 53.7, 118.9, 127.9, 128.1, 128.9, 135.3, 167.0, 175.6, 190.4. MS (*m/z*): 376 [M^+^], 285, 258, 131, 91, 65.

**5,5-dimethyl-2-(piperidin-1-yl)-5,6-dihydrobenzo[*d*]thiazol-7(4H)-one (Table 2, 4d).**

White powder; 89% yield; m.p. = 130-132 ^o^C; ^1^H-NMR (400 MHz, CDCl_3_) δ (ppm): 1.12 (s, 6H, CH_3_), 1.63-1.74 (m, 6H, CH_2_), 2.37 (s, 2H, CH_2_), 2.67 (s, 2H, CH_2_), 3.58 (t, *J =* 5.2 Hz, 4H, CH_2_). ^13^C-NMR (100 MHz, CDCl_3_) δ (ppm): 23.9, 25.2, 28.5, 34.7, 41.5, 49.6, 51.2, 118.2, 167.1, 174.9, 190.1. MS (*m/z*): 264 [M^+^], 235, 208, 182, 149, 111,70.

**5,5-dimethyl-2-(pyrrolidin-1-yl)-5,6-dihydrobenzo[*d*]thiazol-7(4H)-one (Table 2, 4e).**

White powder; 89% yield; m.p. = 150-153 ^o^C; ^1^H-NMR (400 MHz, CDCl_3_) δ (ppm): 1.13 (s, 6H, CH_3_), 2.08-2.11 (m, 4H, CH_2_), 2.39 (s, 2H, CH_2_), 2.72 (s, 2H, CH_2_), 3.54 (bs, 4H, CH_2_). ^13^C-NMR (100 MHz, CDCl_3_) δ (ppm): 25.6, 28.5, 34.8, 41.6, 49.9, 51.2, 118.5, 167.2, 171.5, 190.2. Anal. Calcd for C₁₃H₁₈N₂OS: C, 62.37; H, 7.25; N, 11.19; O, 6.39; S, 12.81. Found: C, 62.49; H, 7.29; N, 11.10; S, 12.70.

**2-(diethylamino)-5,5-dimethyl-5,6-dihydrobenzo[*d*]thiazol-7(4H)-one (Table 2, 4f).**

Light cream powder; 85% yield; m.p. = 89-91 ^o^C; ^1^H-NMR (400 MHz, CDCl_3_) δ (ppm): 1.13 (s, 6H, CH_3_), 1.27 (t, *J* = 7.2 Hz, 6H, CH_3_), 2.38 (s, 2H, CH_2_), 2.68 (s, 2H, CH_2_), 3.56 (q, *J* = 7.2 Hz, 4H, CH_2_). ^13^C-NMR (100 MHz, CDCl_3_) δ (ppm): 12.4, 28.6, 34.7, 41.6, 45.9, 51.2, 118.0, 167.3, 173.8, 190.1. Anal. Calcd for C₁₃H_20_N₂OS: C, 61.87; H, 7.99; N, 11.10; O, 6.34; S, 12.70. Found: C, 61.87; H, 7.99; N, 11.10; S, 12.70.

**2-(dimethylamino)-5,5-dimethyl-5,6-dihydrobenzo[*d*]thiazol-7(4H)-one (Table 2, 4g).**

Cream powder^;^ 84% yield; m.p. = 141-143 ^o^C; ^1^H-NMR (400 MHz, CDCl_3_) δ (ppm): 1.12 (s, 6H, CH_3_), 2.38 (s, 2H, CH_2_), 2.69 (s, 2H, CH_2_), 3.20 (s, 6H, CH_3_). ^13^C-NMR (100 MHz, CDCl_3_) δ (ppm): 28.5, 34.8, 40.4, 41.5, 51.2, 119.0, 167.1, 175.0, 190.2. Anal. Calcd for C₁₁H₁₆N₂OS: C, 58.90; H, 7.19; N, 12.49; O, 7.13; S, 14.29. Found: C, 58.82; H, 7.10; N, 12.58; S, 14.36.

**2-(cyclohexyl(methyl)amino)-5,5-dimethyl-5,6-dihydrobenzo[*d*]thiazol-7(4H)-one Table 2, 4h).**

Brown powder; 87% yield; m.p. = 114-117 ^o^C; ^1^H-NMR (400 MHz, CDCl_3_) δ (ppm): 1.13 (s, 6H, CH_3_), 1.35-1.57 (m, 4H, CH_2_), 1.71-190 (m, 6H, CH_2_), 2.38 (s, 2H, CH_2_), 2.70 (s, 2H, CH_2_), 3.94 (bs, 1H, CH). ^13^C-NMR (100 MHz, CDCl_3_) δ (ppm): 25.3, 25.6, 28.6, 29.8, 32.5, 34.8, 41.6, 51.2, 117.7, 167.1, 174.9, 190.1. Anal. Calcd for C₁₆H₂₄N₂OS: C, 65.71; H, 8.27; N, 9.58; O, 5.47; S, 10.96. Found: C, 65.60; H, 8.33; N, 9.69; S, 11.03.

**5,5-dimethyl-2-morpholino-5,6-dihydrobenzo[*d*]thiazol-7(4H)-one (Table 2, 4i).**

Cream powder; 84% yield; m.p. = 155-158 ^o^C; ^1^H-NMR (400 MHz, CDCl_3_) δ (ppm): 1.14 (s, 6H, CH_3_), 2.40 (s, 2H, CH_2_), 2.70 (s, 2H, CH_2_), 3.61-3.63 (m, 4H, CH_2_), 3.81-3.84 (m, 4H, CH_2_). ^13^C-NMR (100 MHz, CDCl_3_) δ (ppm): 28.5, 34.8, 41.5, 48.3, 51.3, 66.0, 119.1, 166.6, 175.1, 190.4. Anal. Calcd for C₁₃H₁₈N₂O₂S: C, 58.62; H, 6.81; N, 10.52; O, 12.01; S, 12.04. Found: C, 58.71; H, 6.70; N, 10.59; S, 11.92.

**5,5-dimethyl-2-(phenylamino)-5,6-dihydrobenzo[*d*]thiazol-7(4H)-one (Table 2, 4j).**

Light brown powder; 77% yield; m.p. = 179-181 ^o^C; ^1^H-NMR (400 MHz, CDCl_3_) δ (ppm): 1.13 (s, 6H, CH_3_), 2.44 (s, 2H, CH_2_), 2.70 (s, 2H, CH_2_), 7.19-7.49 (m, 5H, Ar), 9.47 (s, 1H, NH). ^13^C-NMR (100 MHz, CDCl_3_) δ (ppm): 28.5, 35.0, 41.2, 51.4, 118.7, 120.5, 125.3, 129.7, 139.0, 165.2, 171.8, 190.8. Anal. Calcd for C₁₅H₁₆N₂OS: C, 66.15; H, 5.92; N, 10.29; O, 5.87; S, 11.77. Found: C, 66.20; H, 6.01; N, 10.20; S, 11.66.

**2-((3,4-dimethylphenyl)amino)-5,5-dimethyl-5,6-dihydrobenzo[*d*]thiazol-7(4H)-one (Table 2, 4k).**

^1^H-NMR (400 MHz, CDCl_3_) δ (ppm): 1.13 (s, 6H, CH_3_), 2.29 (s, 3H, CH_3_), 2.30 (s, 3H, CH_3_), 2.42 (s, 2H, CH_2_), 2.69 (s, 2H, CH_2_), 7.11-7.19 (m, 3H, Ar), 9.12 (bs, 1H, NH). ^13^C-NMR (100 MHz, CDCl_3_) δ (ppm): 19.3, 20.0, 28.5, 35.0, 41.3, 51.3, 118.3, 122.2, 123.6, 130.7, 134.2, 136.6, 138.2, 165.1, 172.5, 190.6. Anal. Calcd for C₁₇H₂₀N₂OS: C, 67.97; H, 6.71; N, 9.32; O, 5.33; S, 10.67. Found: C, 67.88; H, 6.70; N, 9.25; S, 10.76.

**5,5-dimethyl-2-(*o*-tolylamino)-5,6-dihydrobenzo[*d*]thiazol-7(4H)-one (Table 2, 4l).**

Light brown powder; 75% yield; m.p. = 170-172 ^o^C; ^1^H-NMR (400 MHz, CDCl_3_) δ (ppm): 1.07 (s, 6H, CH_3_), 2.34 (s, 3H, CH_3_), 2.37 (s, 2H, CH_2_), 2.53 (s, 2H, CH_2_), 7.22-7.33 (m, 3H, Ar), 7.49 (dd, *J_1_* = 7.8, *J_2_* = 1.6 Hz, 1H, Ar), 9.46 (s, 1H, NH). ^13^C-NMR (100 MHz, CDCl_3_) δ (ppm): 17.9, 28.5, 34.8, 41.0, 51.3, 118.7, 124.3, 127.4, 127.5, 131.5, 132.7, 137.4, 165.4, 174.4, 190.5; MS (*m/z*): 286 [M^+^], 253, 202, 149, 117,70.

**2-((4-chlorophenyl)amino)-5,5-dimethyl-5,6-dihydrobenzo[*d*]thiazol-7(4H)-one (Table 2, 4m).**

Yellow powder; 69% yield; m.p. = 207-209 ^o^C; ^1^H-NMR (400 MHz, CDCl_3_) δ (ppm): 1.15 (s, 6H, CH_3_), 2.45 (s, 2H, CH_2_), 2.72 (s, 2H, CH_2_), 7.35-7.43 (m, 4H, Ar), 9.13 (bs, 1H, NH). ^13^C-NMR (100 MHz, CDCl_3_) δ (ppm): 28.5, 35.0, 41.3, 51.4, 119.3, 121.3, 129.7, 130.0, 137.6, 165.4, 170.8, 191.1. MS (*m/z*): 306 [M^+^], 280, 250, 224, 183, 152, 127, 99, 70.

**2-((4-bromophenyl)amino)-5,5-dimethyl-5,6-dihydrobenzo[*d*]thiazol-7(4H)-one (Table 2, 4n).**

Light brown powder; 74% yield; m.p. = 212-214 ^o^C; ^1^H-NMR (400 MHz, CDCl_3_) δ (ppm): 1.15 (s, 6H, CH_3_), 2.45 (s, 2H, CH_2_), 2.74 (s, 2H, CH_2_), 7.36 (d, *J* = 8.8 Hz, 2H, Ar), 7.52 (d, *J* = 8.8 Hz, 2H, Ar), 8.89 (s, 1H, NH). ^13^C-NMR (100 MHz, CDCl_3_) δ (ppm): 28.5, 35.0, 41.3, 51.4, 117.5, 119.5, 121.4, 132.6, 138.0, 165.3, 170.4, 191.0. MS (*m/z*): 352 [M^+^], 294, 268, 198, 155, 109,70.

**2-(butylamino)-5,5-dimethyl-5,6-dihydrobenzo[*d*]thiazol-7(4H)-one (Table 2, 4o).**

White powder; 68% yield; m.p. = 111-113 ^o^C; ^1^H-NMR (400 MHz, CDCl_3_) δ (ppm): 0.96 (t, *J* = 7.2 Hz, 3H, CH_3_), 1.12 (s, 6H, CH_3_), 1.42 (sext, *J* = 7.2 Hz, 2H, CH_2_), 1.67 (qu, *J* = 7.2 Hz, 2H, CH_2_), 2.39 (s, 2H, CH_2_), 2.65 (s, 2H, CH_2_), 3.30 (t, *J* = 7.2 Hz, 2H, CH_2_), 7.37 (s, 1H, NH). ^13^C-NMR (100 MHz, CDCl_3_) δ (ppm): 13.7, 20.1, 28.5, 30.9, 34.8, 41.4, 46.2, 51.2, 118.0, 166.3, 175.5, 190.1. MS (*m/z*): 252 [M^+^], 209, 182, 140, 114, 70.

**2-(benzylamino)-5,5-dimethyl-5,6-dihydrobenzo[*d*]thiazol-7(4H)-one (Table 2, 4p).**

Light brown powder; 65% yield; m.p. = 178-180 ^o^C; ^1^H-NMR (400 MHz, CDCl_3_) δ (ppm): 1.08 (s, 6H, CH_3_), 2.36 (s, 2H, CH_2_), 2.48 (s, 2H, CH_2_), 4.52 (s, 2H, CH_2_), 7.32-7.42 (m, 5H, Ar), 7.56 (s, 1H, NH). ^13^C-NMR (100 MHz, CDCl_3_) δ (ppm): 28.5, 34.7, 41.0, 50.2, 51.2, 118.6, 127.8, 128.3, 129.0, 136.1, 166.1, 175.2, 190.3. MS (*m/z*): 286 [M^+^], 258, 230, 182, 148, 118, 91, 65.

**2-(benzyl(methyl)amino)-5,6-dihydrobenzo[*d*]thiazol-7(4H)-one (Table 2, 4q).**

Brown powder; 88% yield; m.p. = 104-107 ^o^C; ^1^H-NMR (400 MHz, CDCl_3_) δ (ppm): 2.14 (quint, *J* = 6.4 Hz, 2H, CH_2_), 2.52 (t, *J* = 6.4 Hz, 2H, CH_2_), 2.83 (t, *J* = 6.4 Hz, 2H, CH_2_), 3.10 (s, 3H, CH_3_), 4.77 (s, 2H, CH_2_), 7.25-7.37 (m, 5H, Ar). ^13^C-NMR (100 MHz, CDCl_3_) δ (ppm): 23.0, 27.6, 37.2, 38.3, 56.4, 120.4, 127.7, 128.0, 128.9, 135.5, 168.6, 175.0, 190.8. Anal. Calcd for C₁₅H₁₆N₂OS: C, 66.15; H, 5.92; N, 10.29; O, 5.87; S, 11.77. Found: C, 66.24; H, 6.01; N, 10.22; S, 11.70.

**2-(piperidin-1-yl)-5,6-dihydrobenzo[*d*]thiazol-7(4H)-one (Table 2, 4r).**

Brown powder; 86% yield; m.p. = 89-91 ^o^C; ^1^H-NMR (400 MHz, CDCl_3_) δ (ppm): 1.68-1.75 (m, 6H, CH_2_), 2.13 (quint, *J* = 6.4 Hz, 2H, CH_2_), 2.51 (t, *J* = 6.4 Hz, 2H, CH_2_), 2.80 (t, *J* = 6.4 Hz, 2H, CH_2_), 3.58-3.61 (m, 4H, CH_2_). ^13^C-NMR (100 MHz, CDCl_3_) δ (ppm): 23.0, 24.0, 25.2, 27.6, 37.2, 49.7, 119.7, 168.6, 174.7, 190.8. Anal. Calcd for C₁₂H₁₆N₂OS: C, 60.99; H, 6.82; N, 11.85; O, 6.77; S, 13.57. Found: C, 60.94; H, 6.80; N, 11.95; S, 13.66.

**2-(*p*-tolylamino)-5,6-dihydrobenzo[*d*]thiazol-7(4H)-one (Table 2, 4s).**

Cream powder; 70% yield; m.p. = 217-219 ^o^C; ^1^H-NMR (400 MHz, CDCl_3_) δ (ppm): 2.14 (quint, *J* = 6.4 Hz, 2H, CH_2_), 2.39 (s, 3H, CH_3_), 2.53-2.56 (m, 2H, CH_2_), 2.79 (t, *J* = 6.4 Hz, 2H, CH_2_), 7.22-7.30 (m, 4H, Ar), 9.44 (bs, 1H, NH). ^13^C-NMR (100 MHz, CDCl_3_) δ (ppm): 21.0, 22.9, 27.3, 37.0, 120.0, 121.1, 130.3, 135.5, 136.4, 166.7, 172.3, 191.2. Anal. Calcd for C₁₄H₁₄N₂OS: C, 65.09; H, 5.46; N, 10.84; O, 6.19; S, 12.41. Found: C, 65.15; H, 5.41; N, 10.79; S, 12.51.

**2-((4-methoxyphenyl)amino)-5,6-dihydrobenzo[*d*]thiazol-7(4H)-one (Table 2, 4t).**

Light brown powder; 73% yield; m.p. = 157-159 ^o^C; ^1^H-NMR (400 MHz, CDCl_3_) δ (ppm): 2.13 (quint, *J* = 6.4 Hz, 2H, CH_2_), 2.52-2.55 (m, 2H, CH_2_), 2.75 (t, *J* = 6.4 Hz, 2H, CH_2_), 3.86 (s, 3H, OCH_3_), 6.97 (d, *J* = 8.8 Hz, 2H, Ar), 7.32 (d, *J* = 8.8 Hz, 2H, Ar), 9.45 (s, 1H, NH). ^13^C-NMR (100 MHz, CDCl_3_) δ (ppm): 22.9, 27.3, 37.3, 55.6, 115.0, 119.8, 124.0, 132.0, 157.9, 166.9, 173.7, 191.1. Anal. Calcd for C₁₄H₁₄N₂O₂S: C, 61.29; H, 5.14; N, 10.21; O, 11.66; S, 11.69. Found: C, 61.35; H, 5.10; N, 10.29; S, 11.63.

**2-(benzyl(methyl)amino)-4,6-dimethylthiazolo[4,5-*d*]pyrimidine-5,7(4H,6H)-dione (Table 2, 4y).**

^1^H-NMR (400 MHz, CDCl_3_) δ (ppm): 3.13 (s, 3H, CH_3_), 3.42 (s, 3H, CH_3_), 3.63 (s, 3H, CH_3_), 4.81 (s, 2H, CH_2_), 7.28-7.41 (m, 5H, Ar). ^13^C-NMR (100 MHz, CDCl_3_) δ (ppm): 28.2, 31.8, 38.1, 56.3, 94.6, 127.8, 128.3, 129.0, 135.2, 152.3, 156.0, 157.4, 173.3. Anal. Calcd for C₁₅H₁₆N₄O₂S: C, 56.95; H, 5.10; N, 17.71; O, 10.11; S, 10.13. Found: C, 57.04; H, 5.14; N, 17.69; S, 10.11.

**4,6-dimethyl-2-(methyl(phenyl)amino)thiazolo[4,5-*d*]pyrimidine-5,7(4H,6H)-dione (Table 2, 4z).**

^1^H-NMR (400 MHz, CDCl_3_) δ (ppm): 3.40 (s, 3H, CH_3_), 3.62 (s, 3H, CH_3_), 3.64 (s, 3H, CH_3_), 7.39-7.44 (m, 3H, Ar), 7.49-7.53 (m, 2H, Ar). ^13^C-NMR (100 MHz, CDCl_3_) δ (ppm): 28.2, 31.8, 40.7, 95.2, 125.7, 128.6, 130.4, 144.1, 152.3, 155.6, 157.4, 173.4. Anal. Calcd for C₁₄H₁₄N₄O₂S: C, 55.62; H, 4.67; N, 18.53; O, 10.58; S, 10.60. Found: C, 55.53; H, 4.73; N, 18.60; S, 10.59.

**2-(propylamino)-4H-chromeno[4,3-*d*]thiazol-4-one ((Table 2, 4aa).**

Light brown powder; 85% yield; m.p. = 146-148 ^o^C; ^1^H-NMR (400 MHz, CDCl_3_) δ (ppm): 1.7 (t, *J* = 7.2 Hz, 3H, CH_3_), 1.79 (sext, *J* = 7.2 Hz, 2H, CH_2_), 3.42 (q, *J* = 7.2 Hz, 2H, CH_2_), 6.38 (s, 1H, NH), 7.34 (dt, *J*_1_ = 7.6, *J*_2_ = 1.2 Hz, 1H, Ar), 7.43 (dd, *J*_1_ = 8.0, *J*_2_ = 1.2 Hz, 1H, Ar), 7.54 (dt, *J*_1_ = 7.2, *J*_2_ = 1.6 Hz, 1H, Ar). ^13^C-NMR (100 MHz, CDCl_3_) δ (ppm): 11.4, 22.3, 48.2, 108.7, 116.7, 117.2, 124.3, 131.0, 153.7, 157.2, 158.8, 175.7. Anal. Calcd for C₁₃H₁₂N₂O₂S: C, 59.98; H, 4.65; N, 10.76; O, 12.29; S, 12.32. Found: C, 60.01; H, 4.69; N, 10.80; S, 12.25.


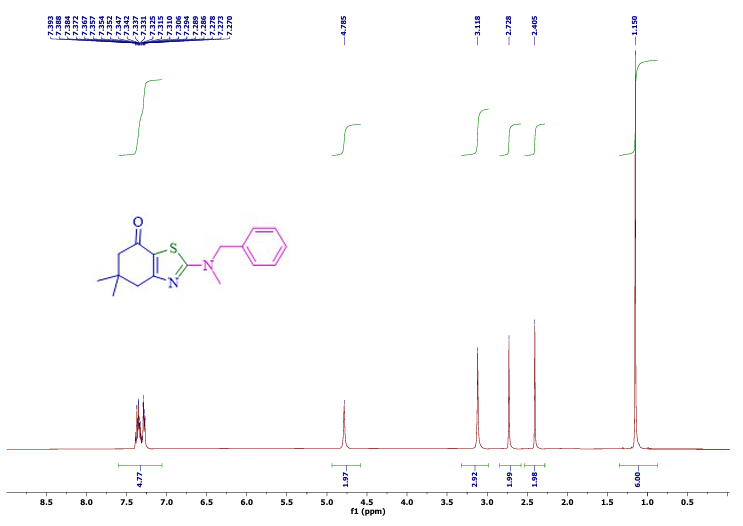


**^1^H-NMR of 2-(benzyl(methyl)amino)-5,5-dimethyl-5,6-dihydrobenzo[*d*]thiazol-7(4H)-one (Table 2, 4a).**


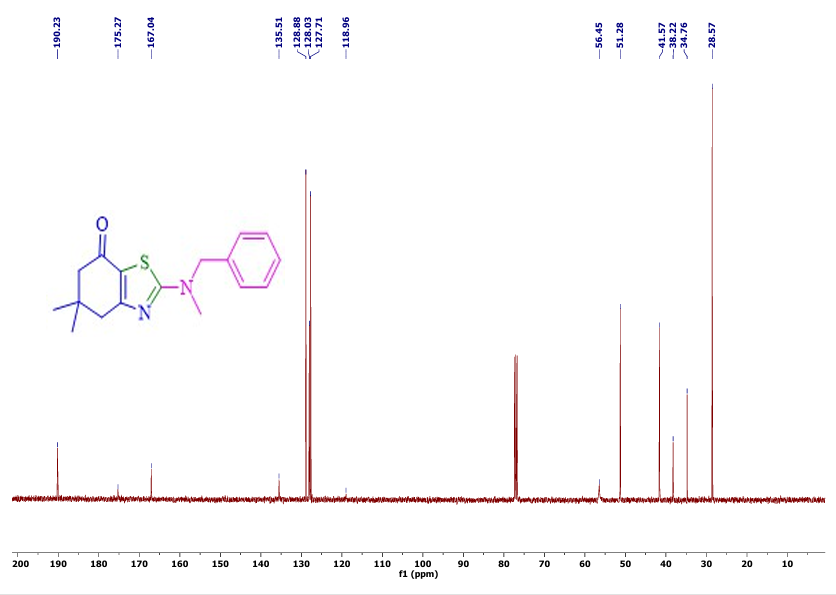


**^13^C-NMR of 2-(benzyl(methyl)amino)-5,5-dimethyl-5,6-dihydrobenzo[*d*]thiazol-7(4H)-one (Table 2, 4a).**


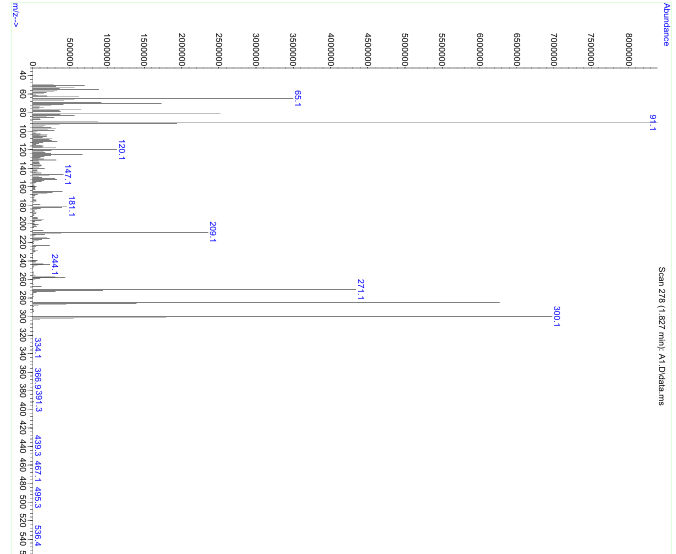


C_17_H_20_N_2_OS (**4a**)

MS (*m/z*) 300


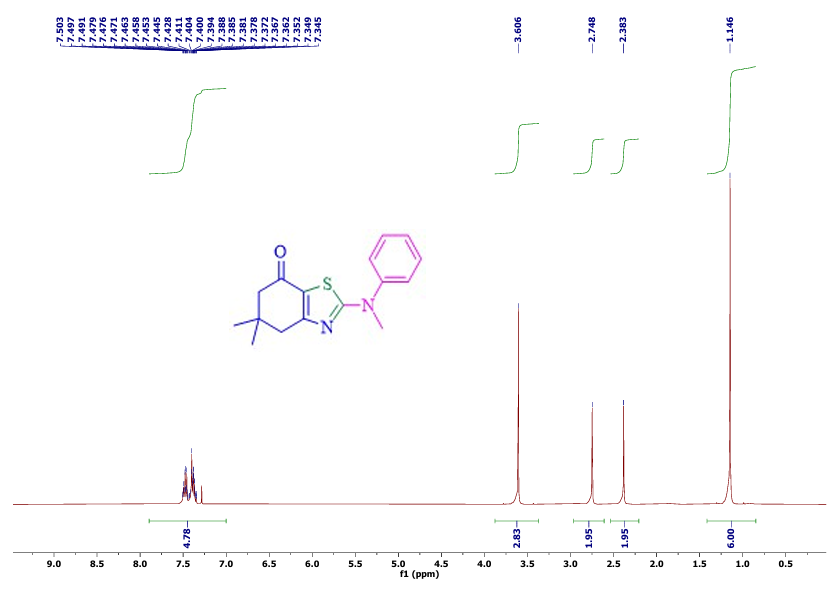


**^1^H-NMR of 5,5-dimethyl-2-(methyl(phenyl)amino)-5,6-dihydrobenzo[*d*]thiazol-7(4H)-one (Table 2, 4b).**


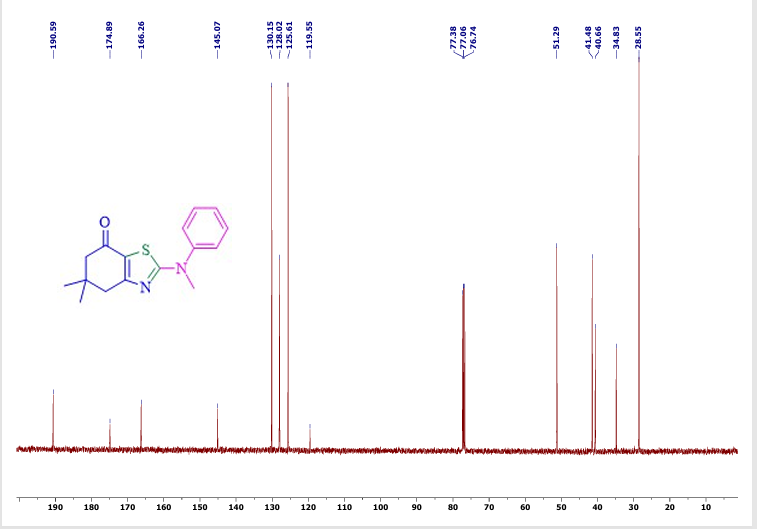


**^13^C-NMR of 5,5-dimethyl-2-(methyl(phenyl)amino)-5,6-dihydrobenzo[*d*]thiazol-7(4H)-one (Table 2, 4b).**


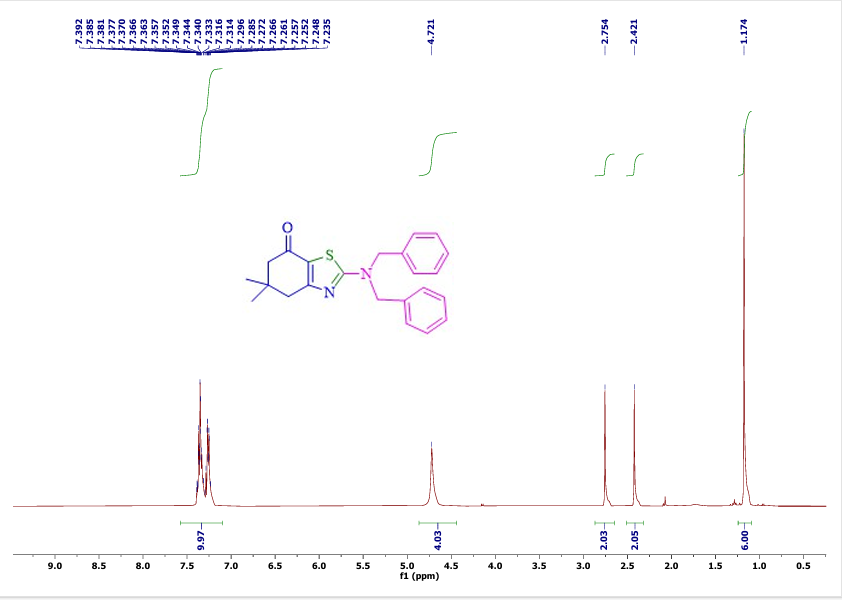


**^1^H-NMR of 2-(dibenzylamino)-5,5-dimethyl-5,6-dihydrobenzo[*d*]thiazol-7(4H)-one (Table 2, 4c).**


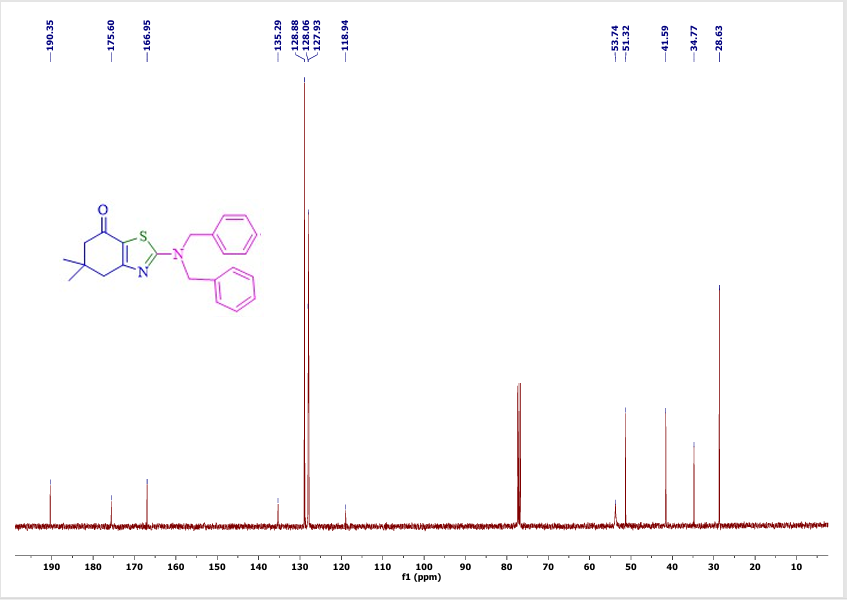


**^13^C-NMR of 2-(dibenzylamino)-5,5-dimethyl-5,6-dihydrobenzo[*d*]thiazol-7(4H)-one (Table 2, 4c).**


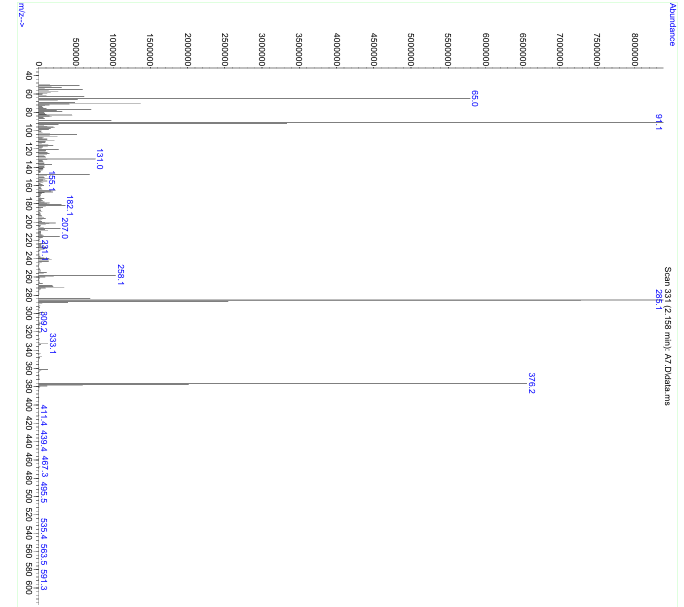


C_23_H_24_N_2_OS (**4c**)

MS (*m/z*): 376


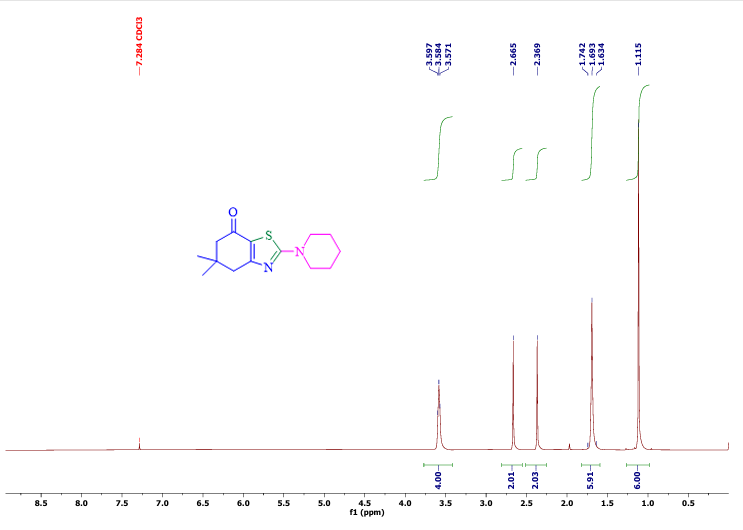


**^1^H-NMR of 5,5-dimethyl-2-(piperidin-1-yl)-5,6-dihydrobenzo[*d*]thiazol-7(4H)-one (Table 2, 4d).**


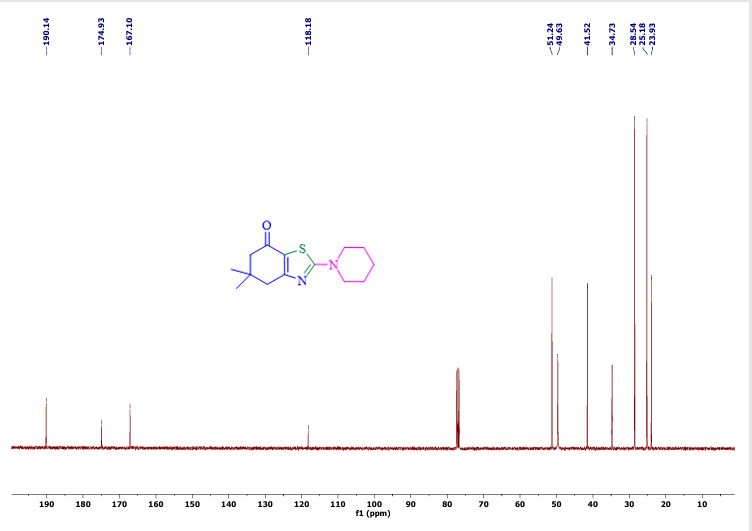


**^13^C-NMR of 5,5-dimethyl-2-(piperidin-1-yl)-5,6-dihydrobenzo[*d*]thiazol-7(4H)-one (Table 2, 4d).**


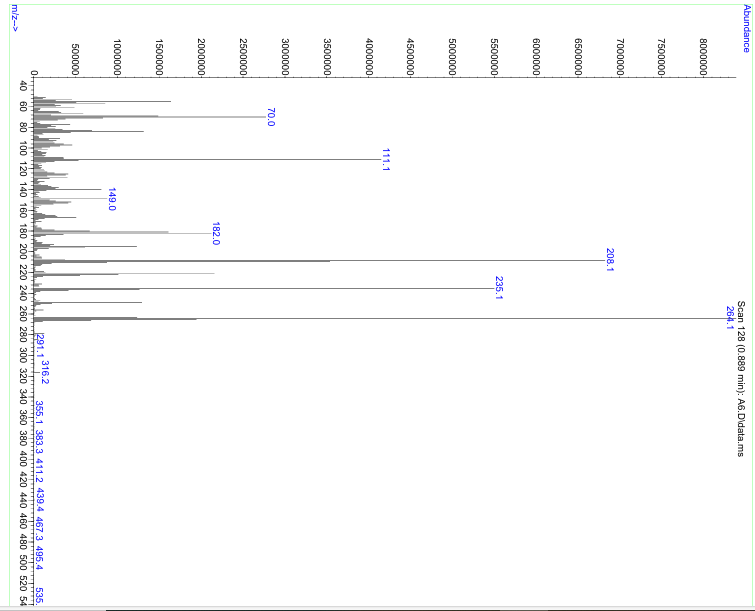


C_14_H_20_N_2_OS **(4d)**

MS (*m/z*): 264


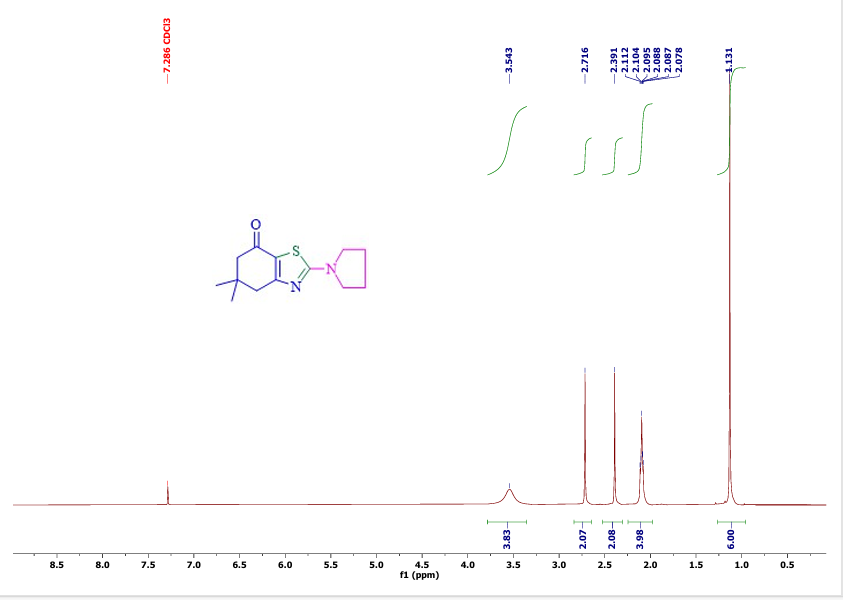


**^1^H-NMR of 5,5-dimethyl-2-(pyrrolidin-1-yl)-5,6-dihydrobenzo[*d*]thiazol-7(4H)-one (Table 2, 4e).**


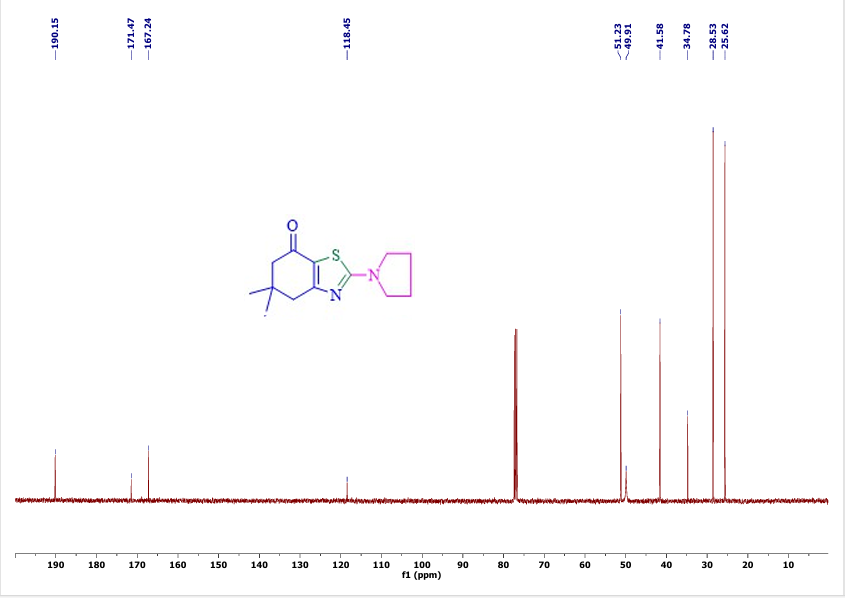


**^13^C-NMR of 5,5-dimethyl-2-(pyrrolidin-1-yl)-5,6-dihydrobenzo[*d*]thiazol-7(4H)-one (Table 2, 4e).**


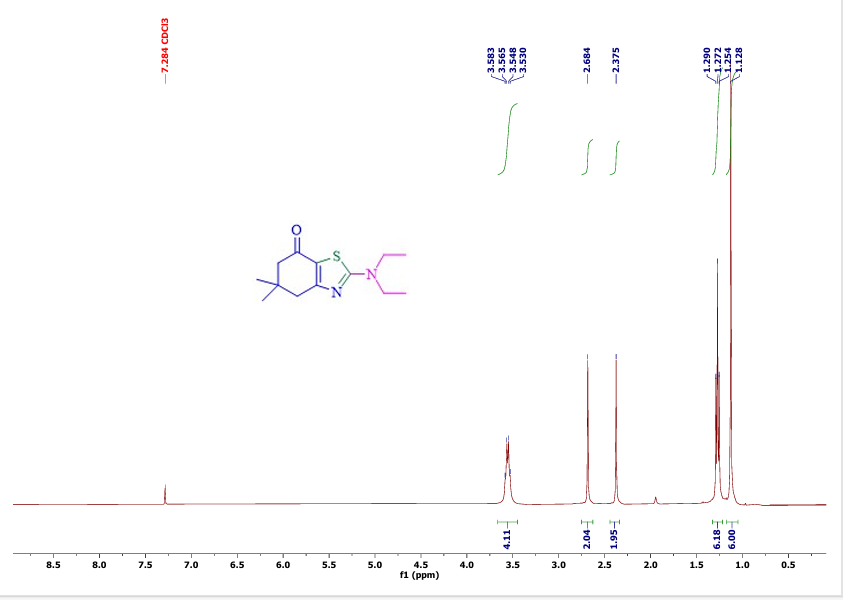


**^1^H-NMR of 2-(diethylamino)-5,5-dimethyl-5,6-dihydrobenzo[*d*]thiazol-7(4H)-one (Table 2, 4f).**


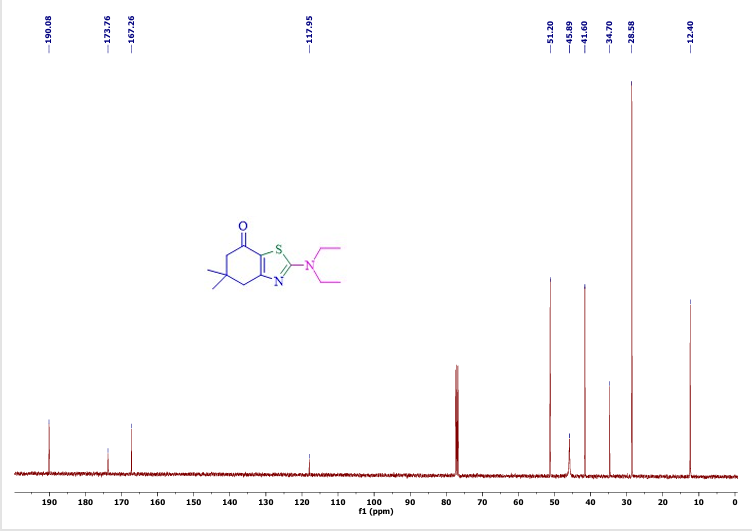


**^13^C-NMR of 2-(diethylamino)-5,5-dimethyl-5,6-dihydrobenzo[*d*]thiazol-7(4H)-one (Table 2, 4f).**


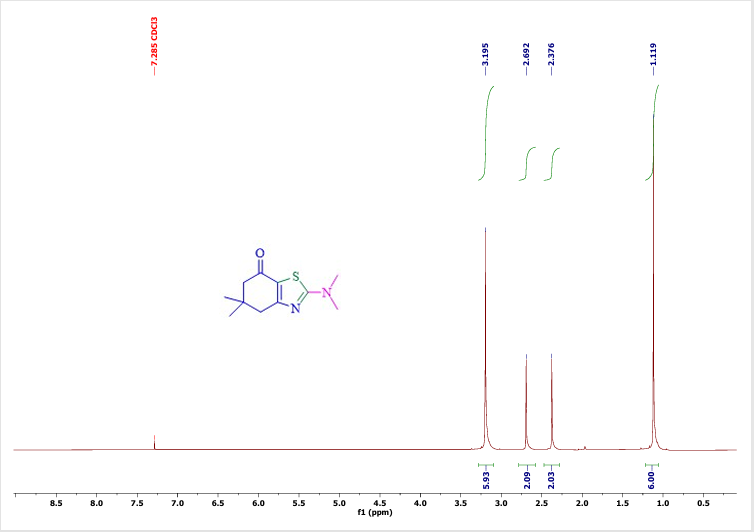


**^1^H-NMR of 2-(dimethylamino)-5,5-dimethyl-5,6-dihydrobenzo[*d*]thiazol-7(4H)-one (Table 2, 4g).**


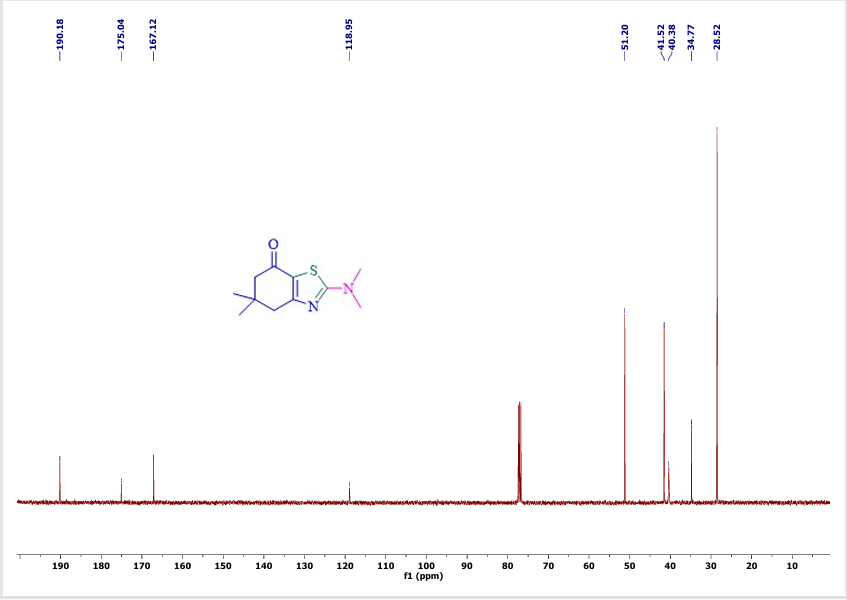


**^13^C-NMR of 2-(dimethylamino)-5,5-dimethyl-5,6-dihydrobenzo[*d*]thiazol-7(4H)-one (Table 2, 4g).**


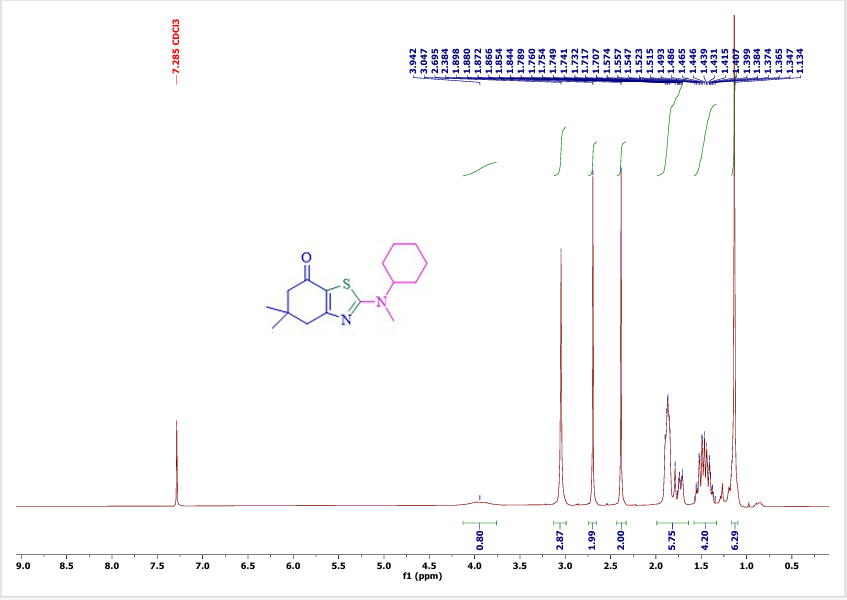


**^1^H-NMR of 2-(cyclohexyl(methyl)amino)-5,5-dimethyl-5,6-dihydrobenzo[*d*]thiazol-7(4H)-one Table 2, 4h).**


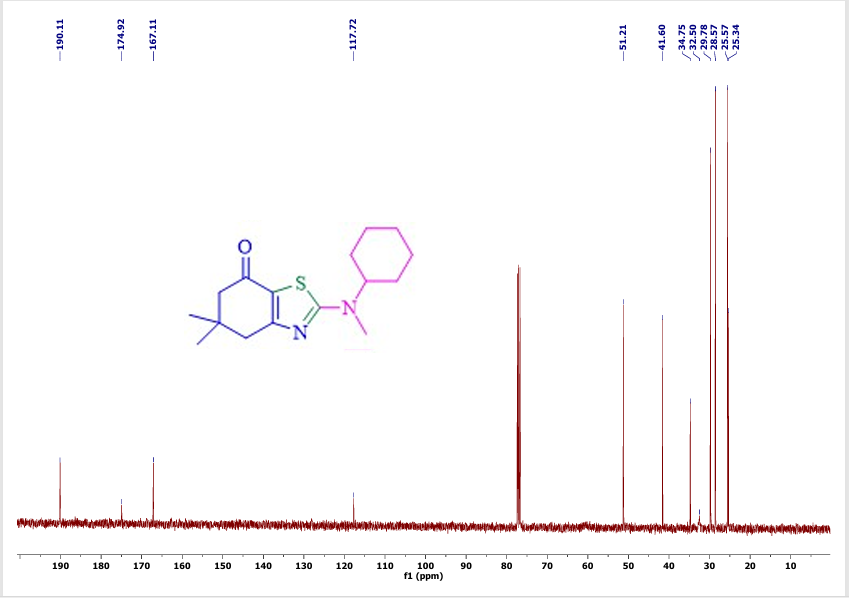


**^13^C-NMR of 2-(cyclohexyl(methyl)amino)-5,5-dimethyl-5,6-dihydrobenzo[*d*]thiazol-7(4H)-one Table 2, 4h).**


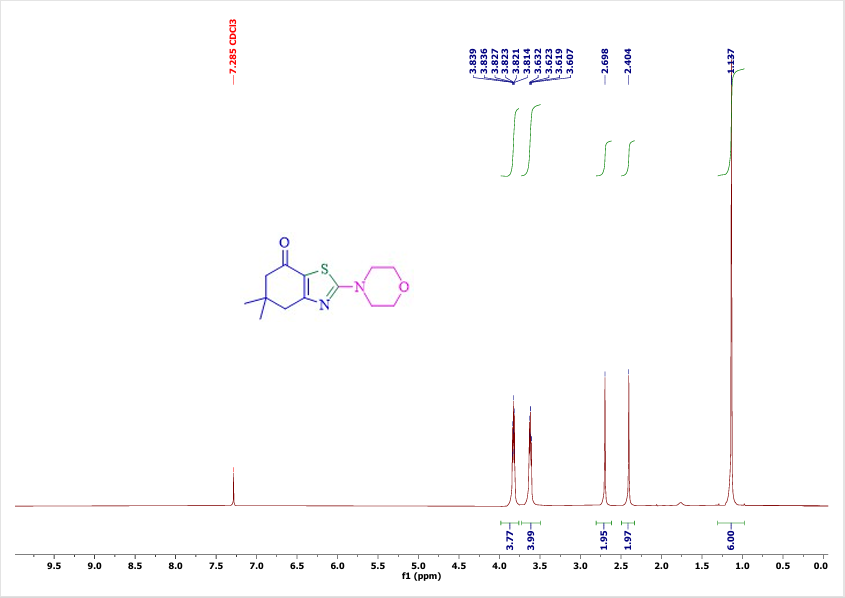


**^1^H-NMR of 5,5-dimethyl-2-morpholino-5,6-dihydrobenzo[*d*]thiazol-7(4H)-one (Table 2, 4i).**


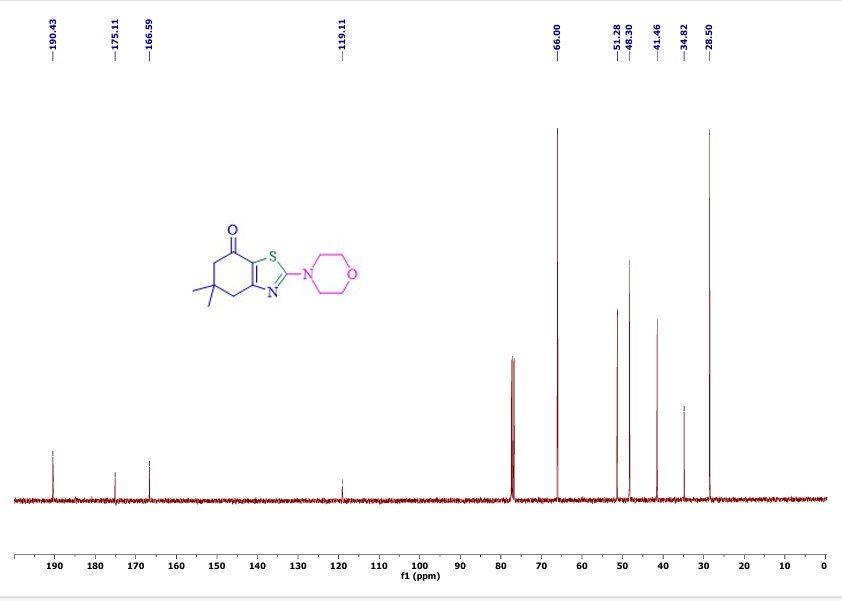


**^13^C-NMR of 5,5-dimethyl-2-morpholino-5,6-dihydrobenzo[*d*]thiazol-7(4H)-one (Table 2, 4i).**


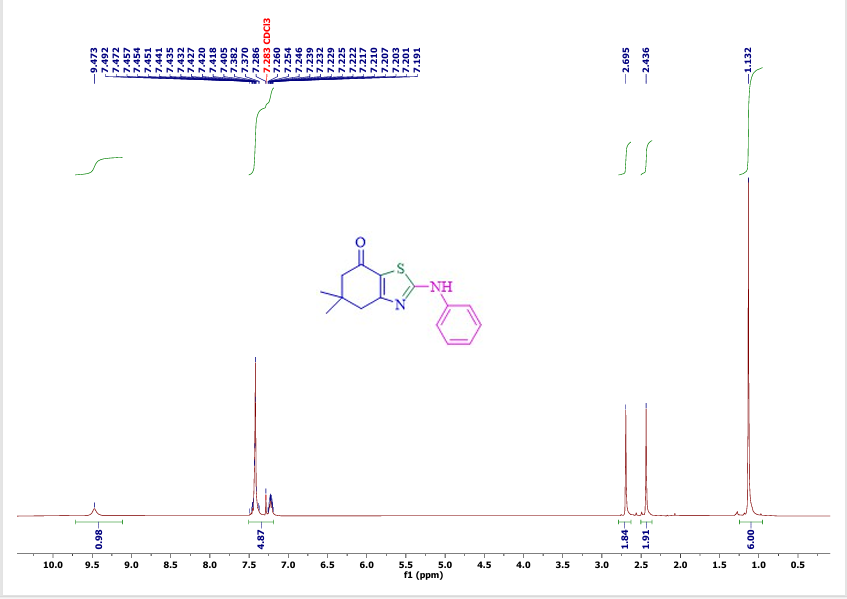


**^1^H-NMR of 5,5-dimethyl-2-(phenylamino)-5,6-dihydrobenzo[*d*]thiazol-7(4H)-one (Table 2, 4j).**


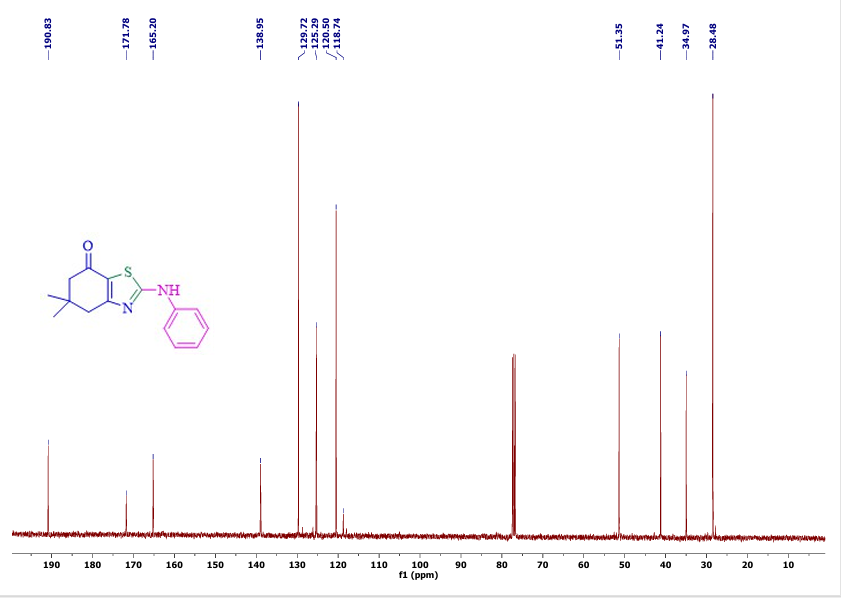


**^13^C-NMR of 5,5-dimethyl-2-(phenylamino)-5,6-dihydrobenzo[*d*]thiazol-7(4H)-one (Table 2, 4j).**


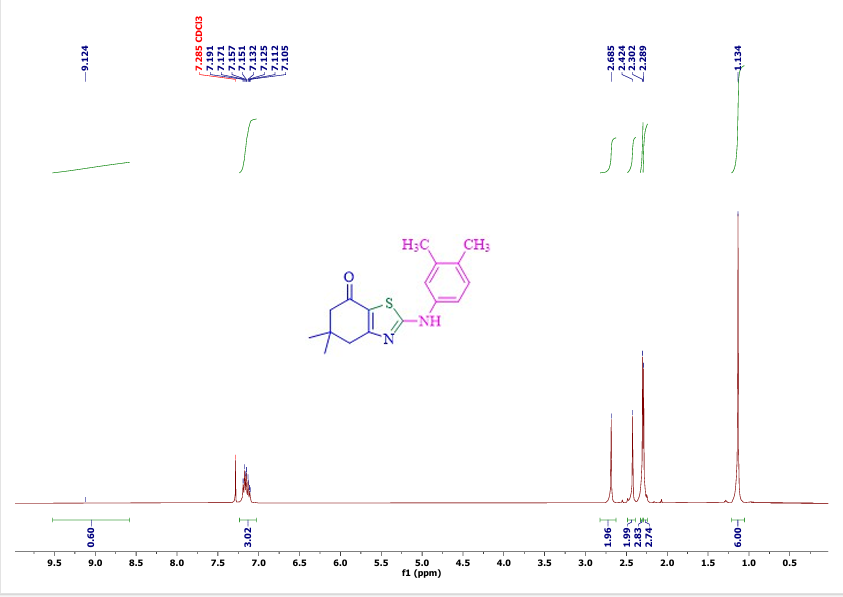


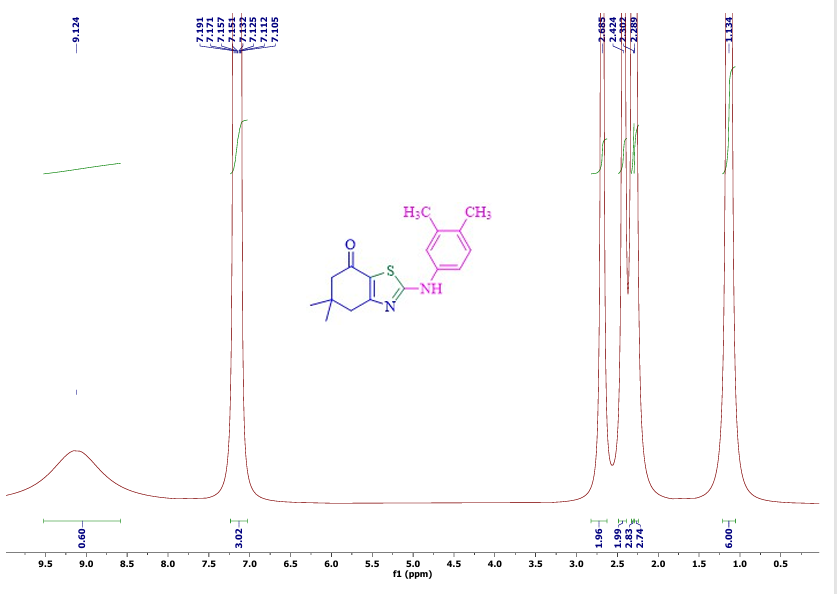


**^1^H-NMR of 2-((3,4-dimethylphenyl)amino)-5,5-dimethyl-5,6-dihydrobenzo[d]thiazol-7(4H)-one (Table 2, 4k).**


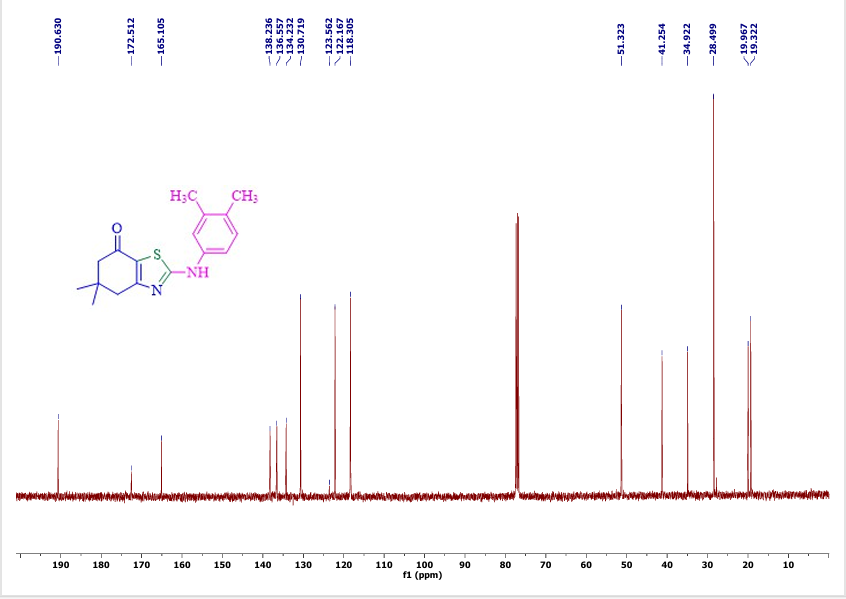


**^13^C-NMR of 2-((3,4-dimethylphenyl)amino)-5,5-dimethyl-5,6-dihydrobenzo[*d*]thiazol-7(4H)-one (Table 2, 4k).**


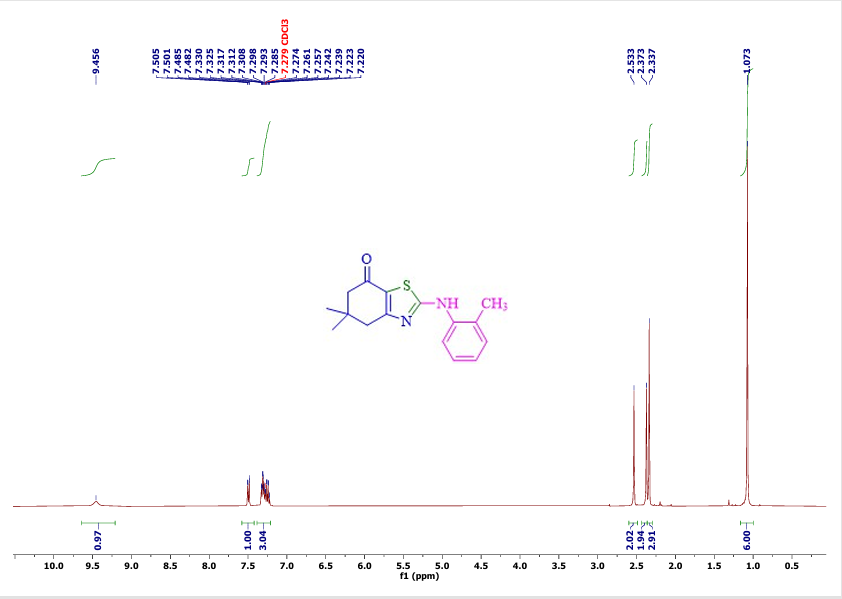


**^1^H-NMR of 5,5-dimethyl-2-(*o*-tolylamino)-5,6-dihydrobenzo[*d*]thiazol-7(4H)-one (Table 2, 4l).**


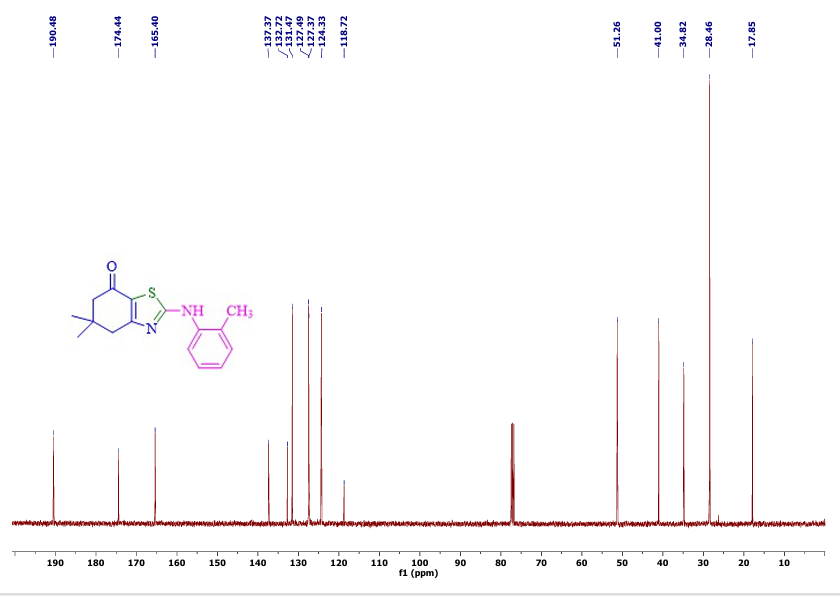


**^13^C-NMR of 5,5-dimethyl-2-(*o*-tolylamino)-5,6-dihydrobenzo[*d*]thiazol-7(4H)-one (Table 2, 4l).**


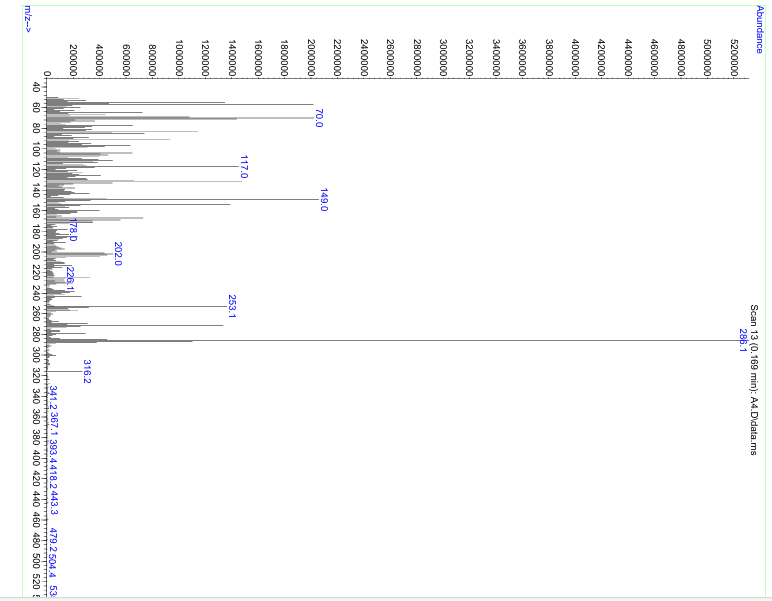


C_16_H_18_N_2_OS **(4l)**

MS (*m/z*): 286


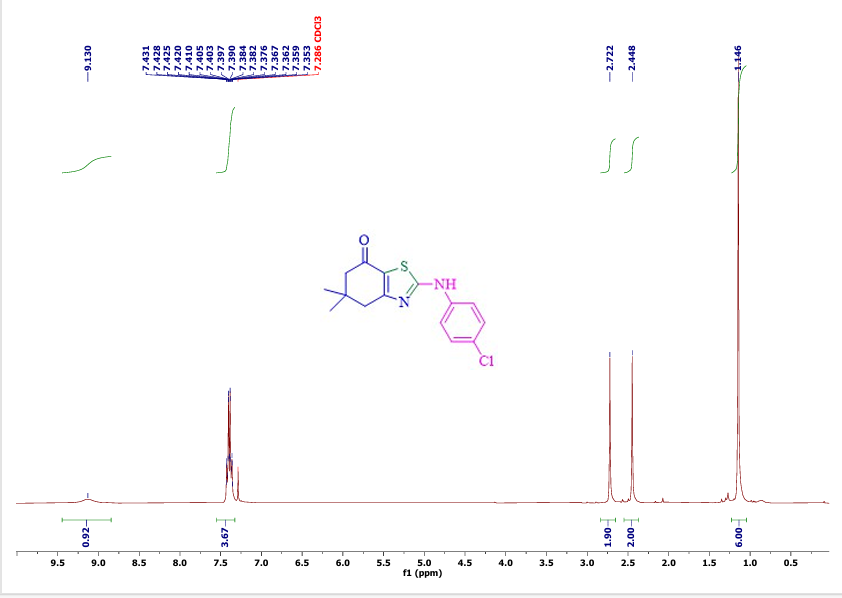


**^1^H-NMR of 2-((4-chlorophenyl)amino)-5,5-dimethyl-5,6-dihydrobenzo[*d*]thiazol-7(4H)-one (Table 2, 4m).**


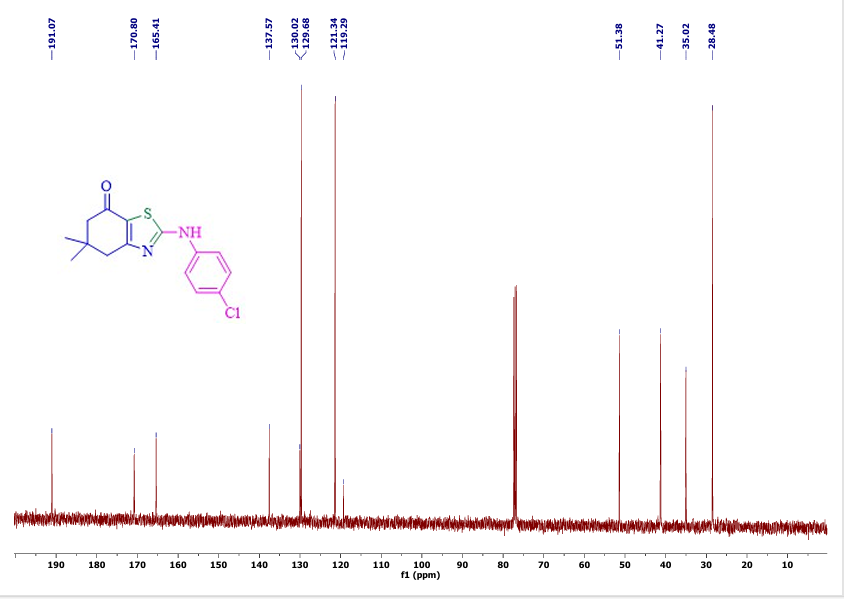


**^13^C-NMR of 2-((4-chlorophenyl)amino)-5,5-dimethyl-5,6-dihydrobenzo[*d*]thiazol-7(4H)-one (Table 2, 4m).**


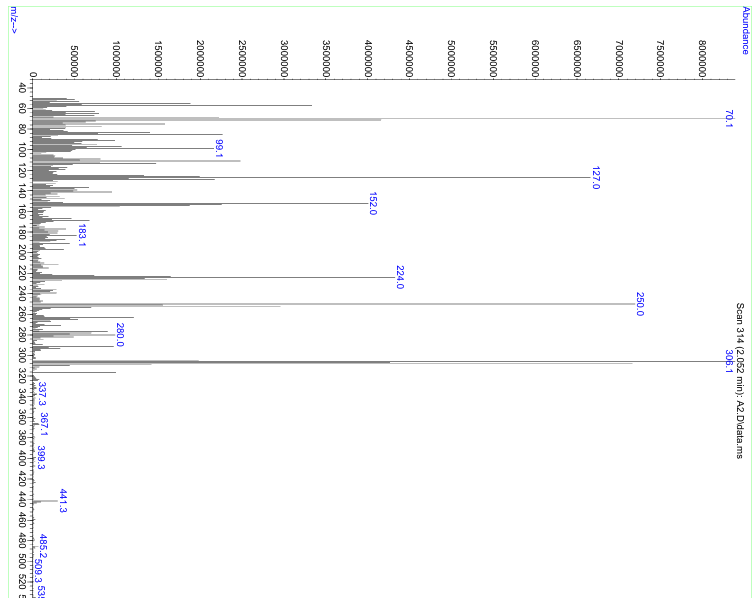


C_15_H_15_ClN_2_OS **(4m)**

MS (*m/z*): 306


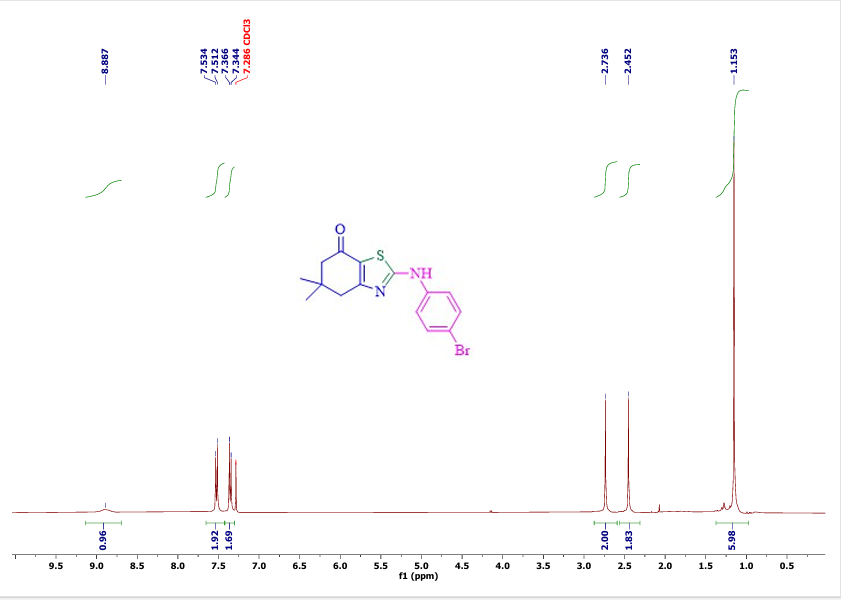


**^1^H-NMR of 2-((4-bromophenyl)amino)-5,5-dimethyl-5,6-dihydrobenzo[*d*]thiazol-7(4H)-one (Table 2, 4n).**


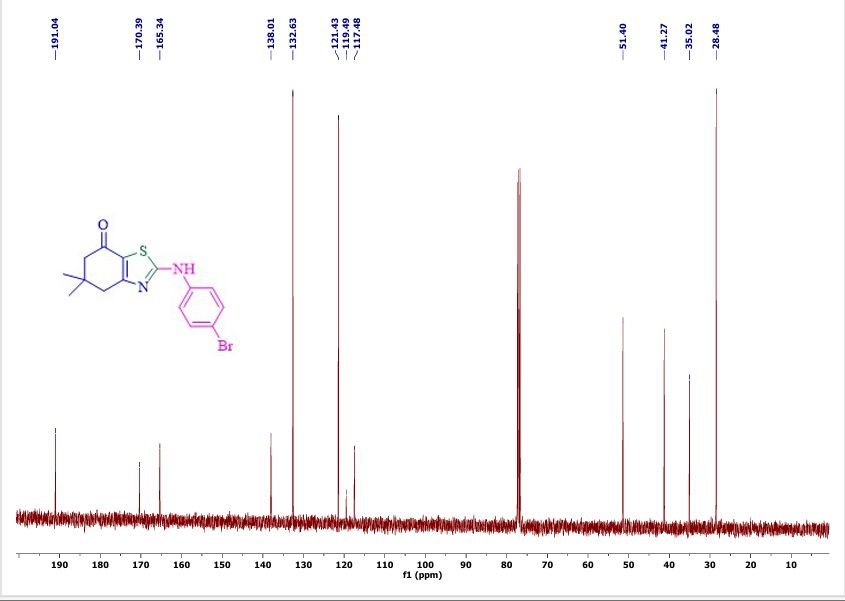


**^13^C-NMR of 2-((4-bromophenyl)amino)-5,5-dimethyl-5,6-dihydrobenzo[*d*]thiazol-7(4H)-one (Table 2, 4n).**


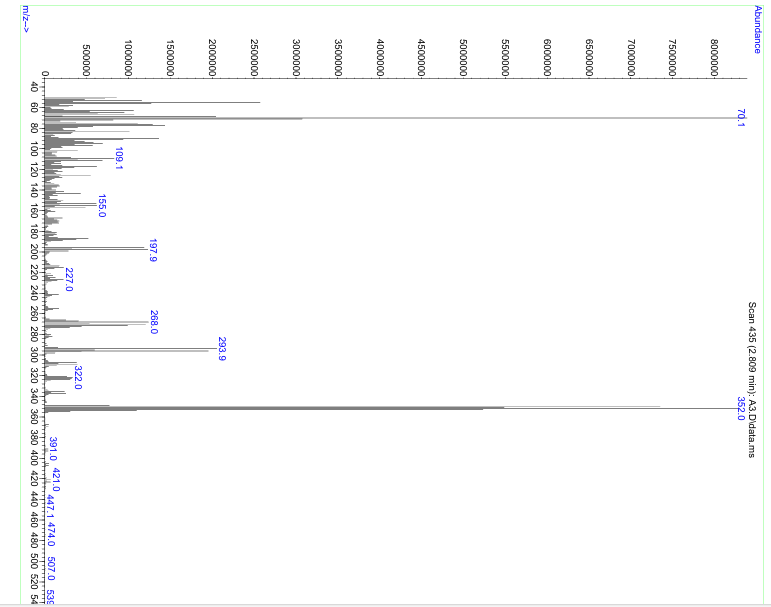


C_15_H_15_BrN_2_OS **(4n)**

MS (*m/z*): 352


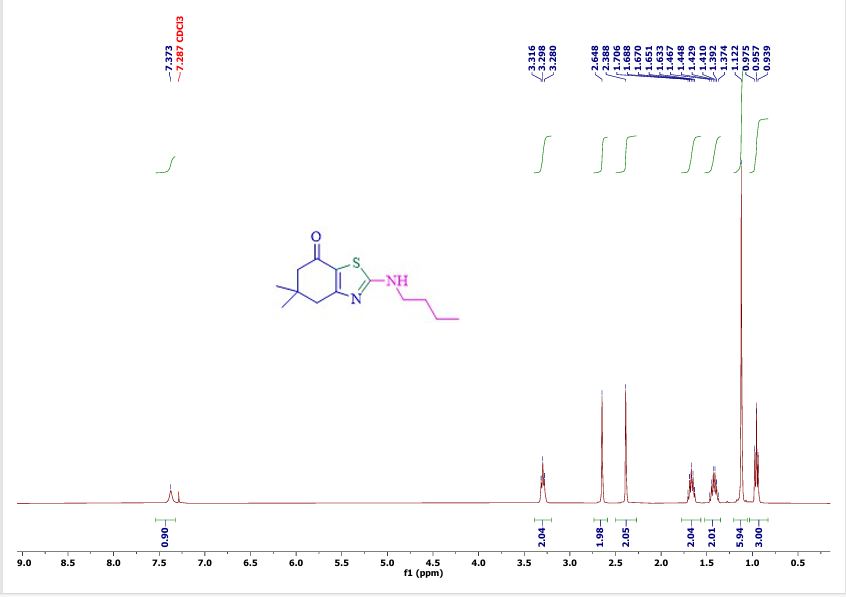


**^1^H-NMR of 2-(butylamino)-5,5-dimethyl-5,6-dihydrobenzo[*d*]thiazol-7(4H)-one (Table 2, 4o).**


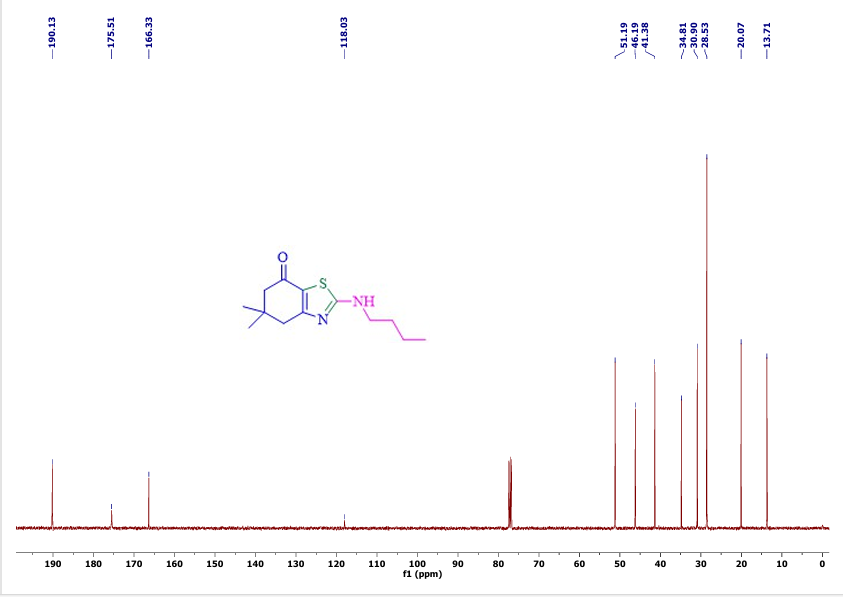


**^13^C-NMR of 2-(butylamino)-5,5-dimethyl-5,6-dihydrobenzo[*d*]thiazol-7(4H)-one (Table 2, 4o).**


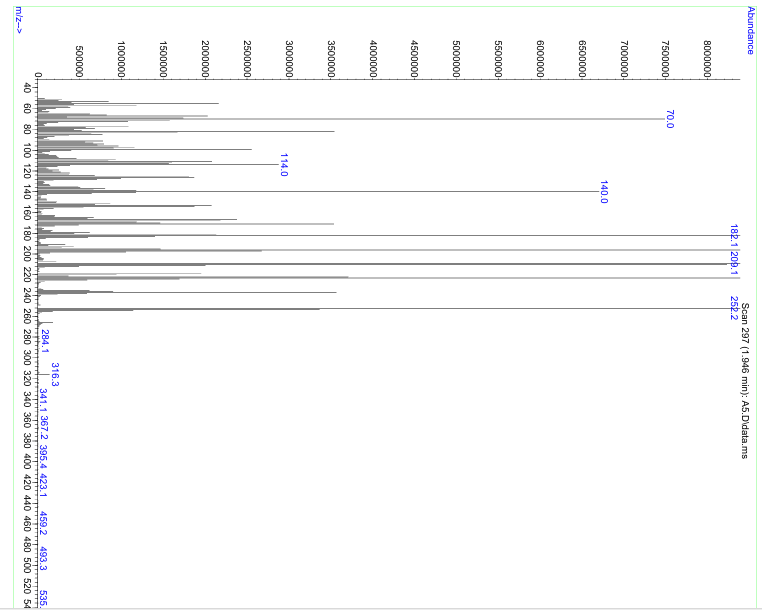


C_13_H_20_N_2_OS **(4o)**

MS (*m/z*): 252


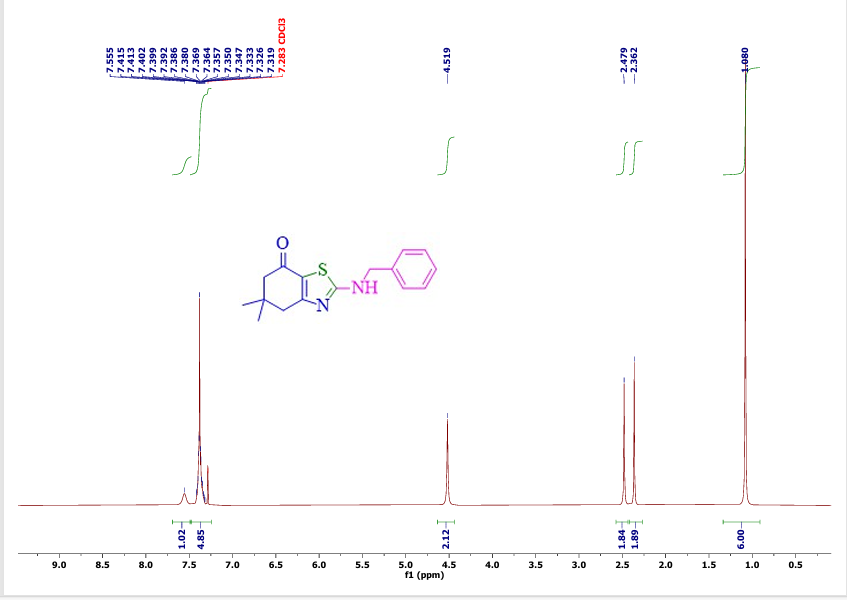


**^1^H-NMR of 2-(benzylamino)-5,5-dimethyl-5,6-dihydrobenzo[*d*]thiazol-7(4H)-one (Table 2, 4p).**


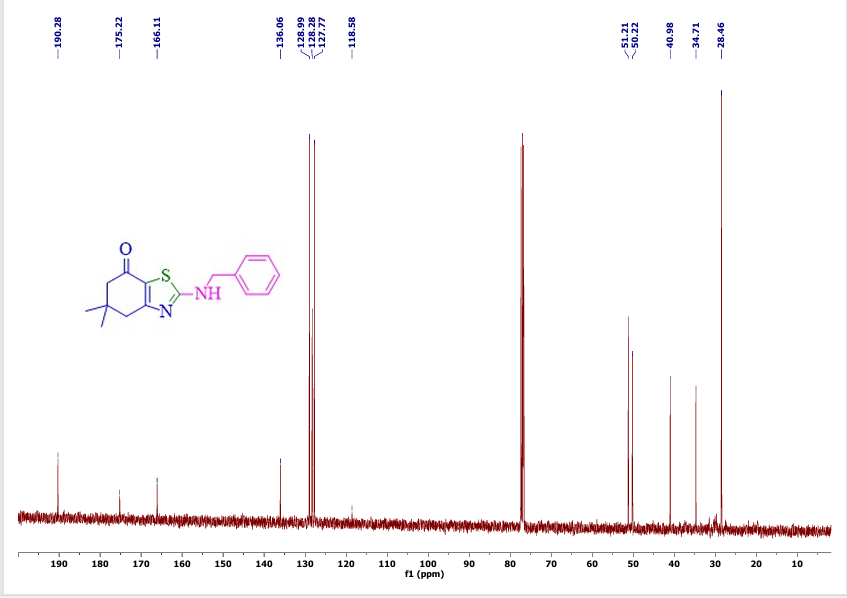


**^13^C-NMR of 2-(benzylamino)-5,5-dimethyl-5,6-dihydrobenzo[*d*]thiazol-7(4H)-one (Table 2, 4p).**


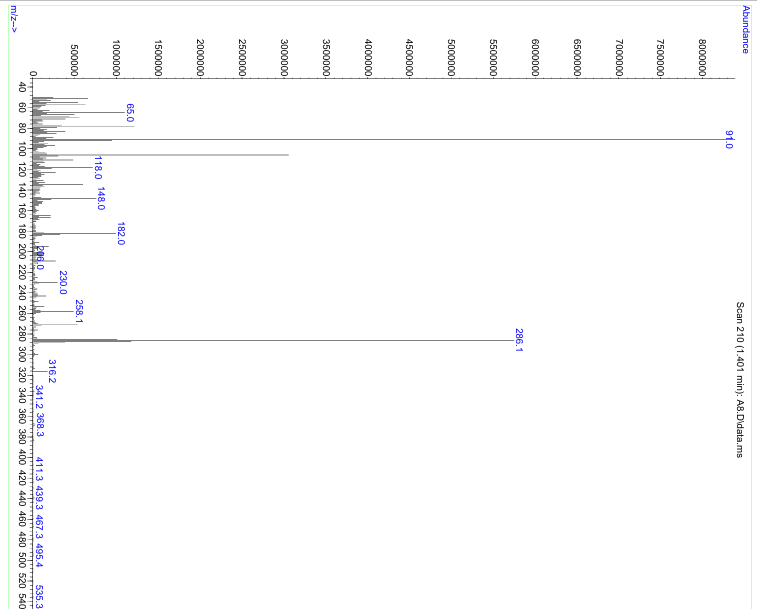


C_16_H_18_N_2_OS **(4p)**

MS (*m/z*): 286


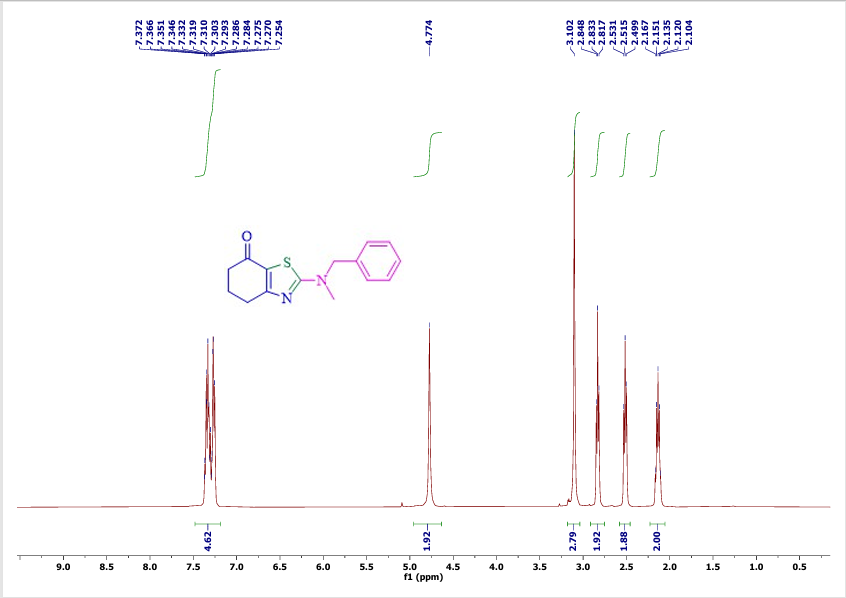


**^1^H-NMR of 2-(benzyl(methyl)amino)-5,6-dihydrobenzo[*d*]thiazol-7(4H)-one (Table 2, 4q).**


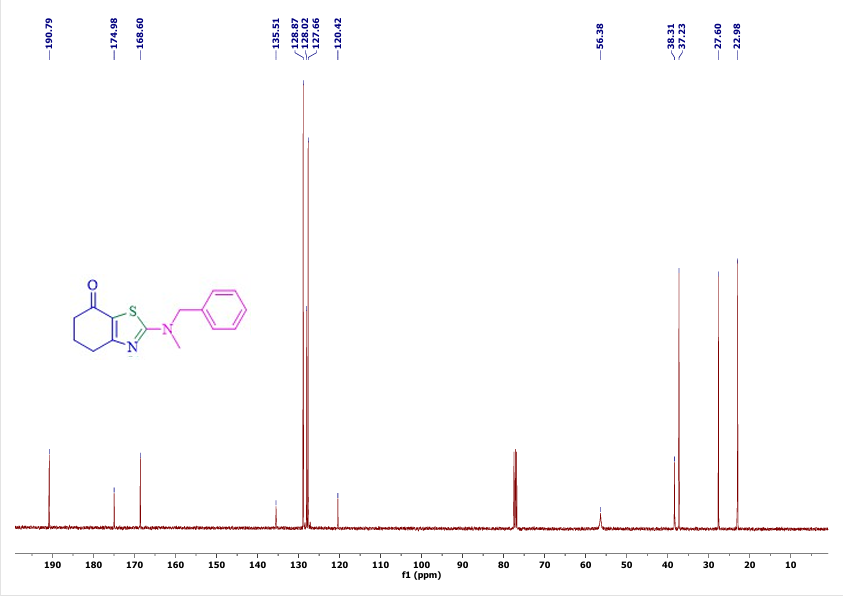


**^13^C-NMR of 2-(benzyl(methyl)amino)-5,6-dihydrobenzo[*d*]thiazol-7(4H)-one (Table 2, 4q).**


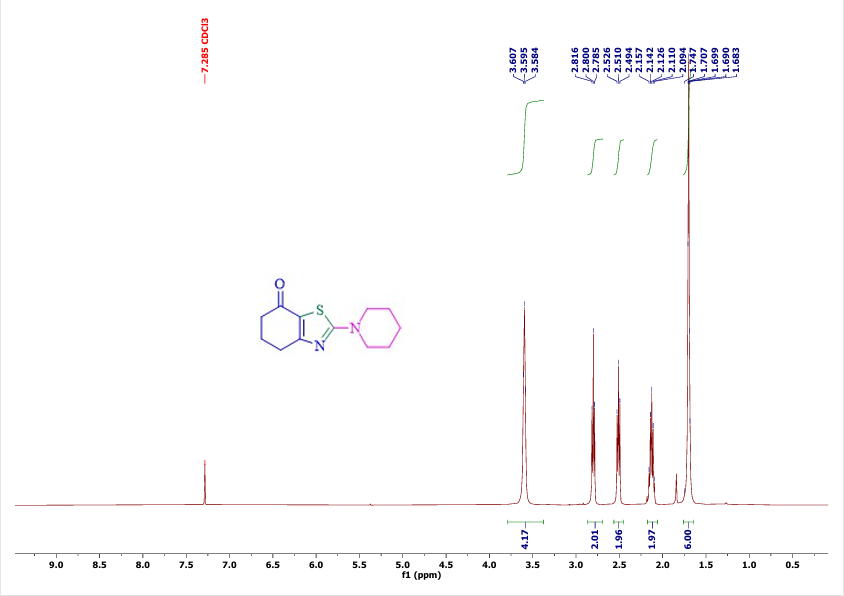


**^1^H-NMR of 2-(piperidin-1-yl)-5,6-dihydrobenzo[*d*]thiazol-7(4H)-one (Table 2, 4r).**


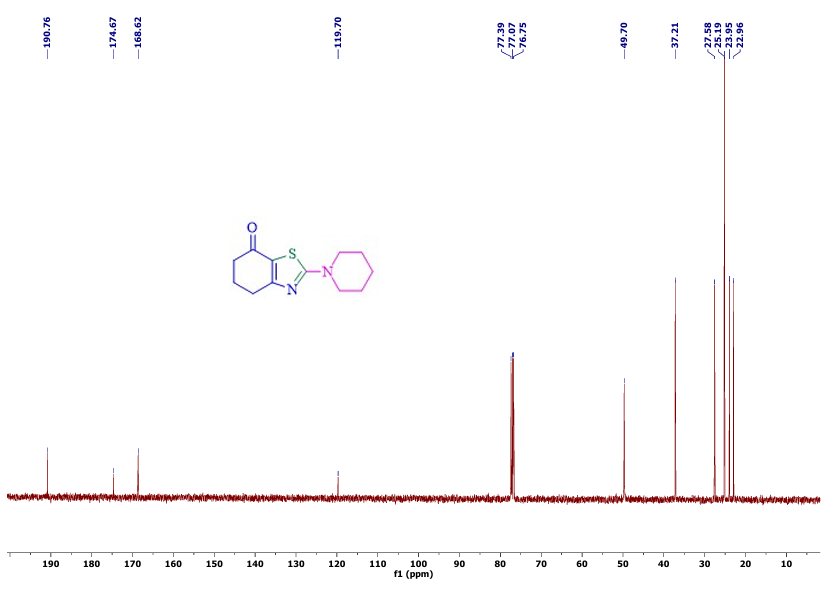


**^13^C-NMR of 2-(piperidin-1-yl)-5,6-dihydrobenzo[*d*]thiazol-7(4H)-one (Table 2, 4r).**


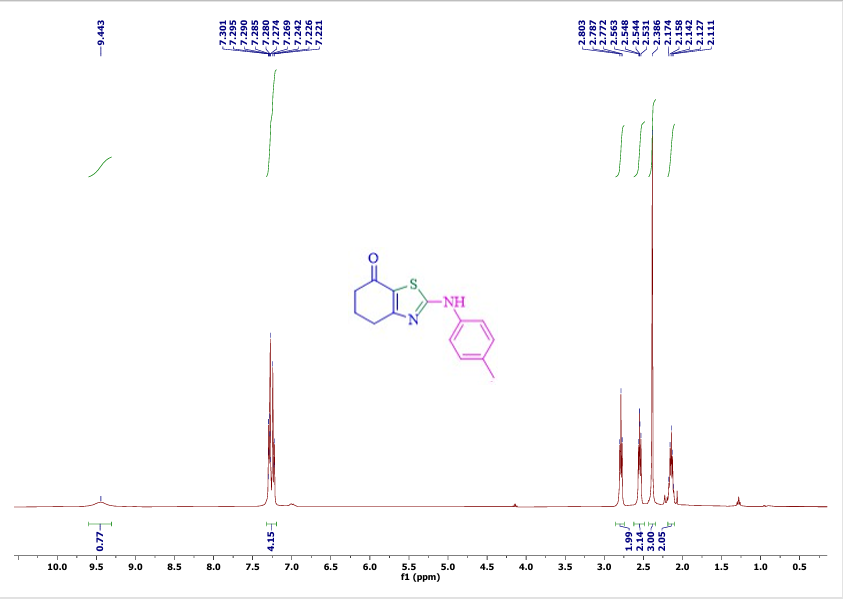


**^1^H-NMR of 2-(*p*-tolylamino)-5,6-dihydrobenzo[*d*]thiazol-7(4H)-one (Table 2, 4s).**


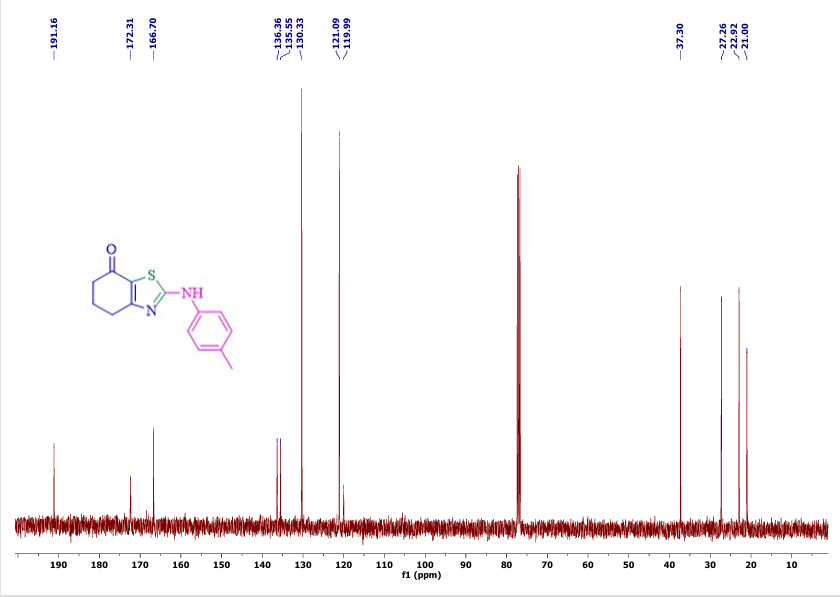


**^13^C-NMR of 2-(*p*-tolylamino)-5,6-dihydrobenzo[*d*]thiazol-7(4H)-one (Table 2, 4s).**


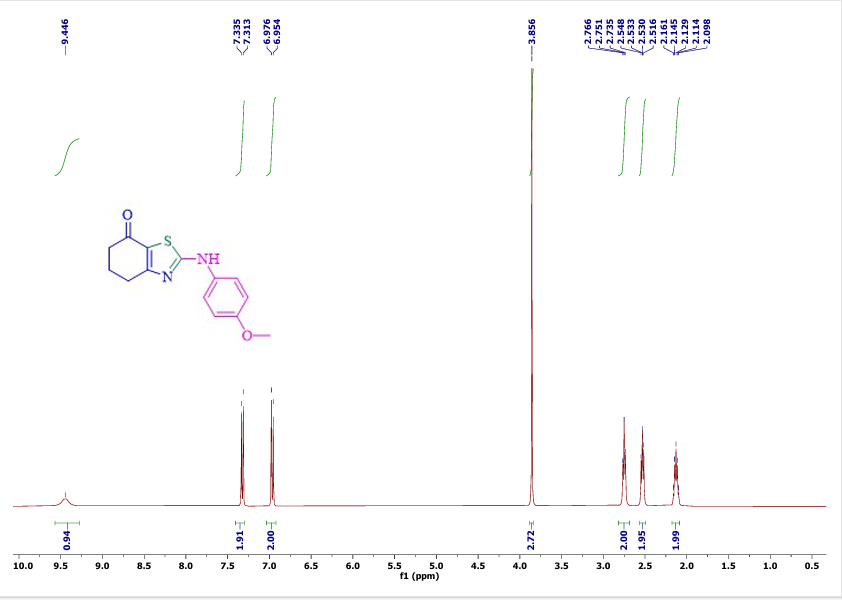


**^1^H-NMR of 2-((4-methoxyphenyl)amino)-5,6-dihydrobenzo[*d*]thiazol-7(4H)-one (Table 2, 4t).**


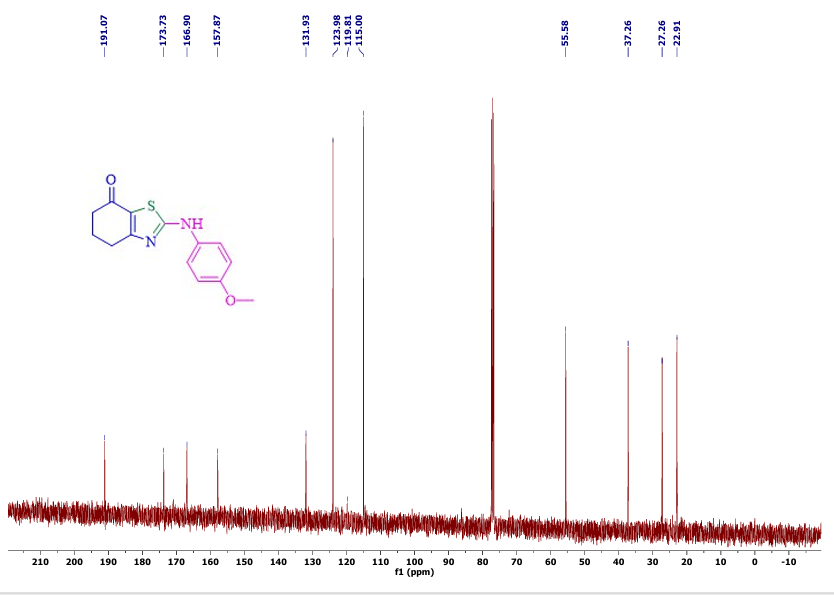


**^13^C-NMR of 2-((4-methoxyphenyl)amino)-5,6-dihydrobenzo[*d*]thiazol-7(4H)-one (Table 2, 4t).**


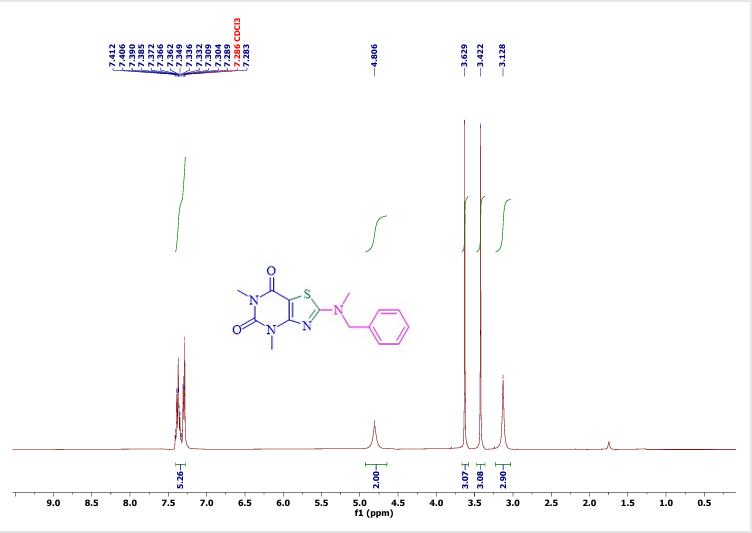


**^1^H-NMR of 2-(benzyl(methyl)amino)-4,6-dimethylthiazolo[4,5-*d*]pyrimidine-5,7(4H,6H)-dione (Table 2, 4y).**


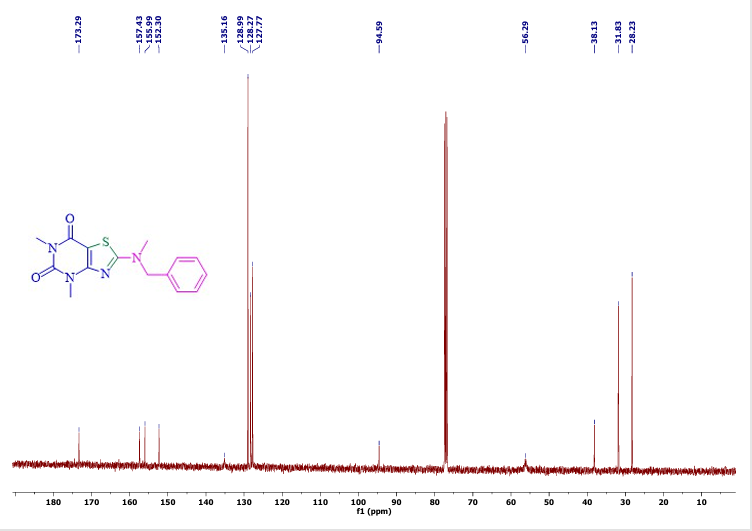


**^13^C-NMR of 2-(benzyl(methyl)amino)-4,6-dimethylthiazolo[4,5-*d*]pyrimidine-5,7(4H,6H) -dione (Table 2, 4y).**


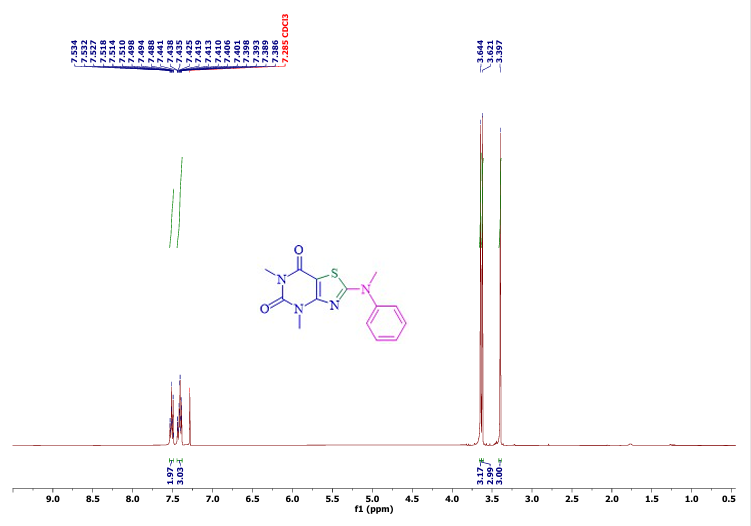


**^1^H-NMR of 4,6-dimethyl-2-(methyl(phenyl)amino)thiazolo[4,5-*d*]pyrimidine-5,7(4H,6H) -dione (Table 2, 4z).**


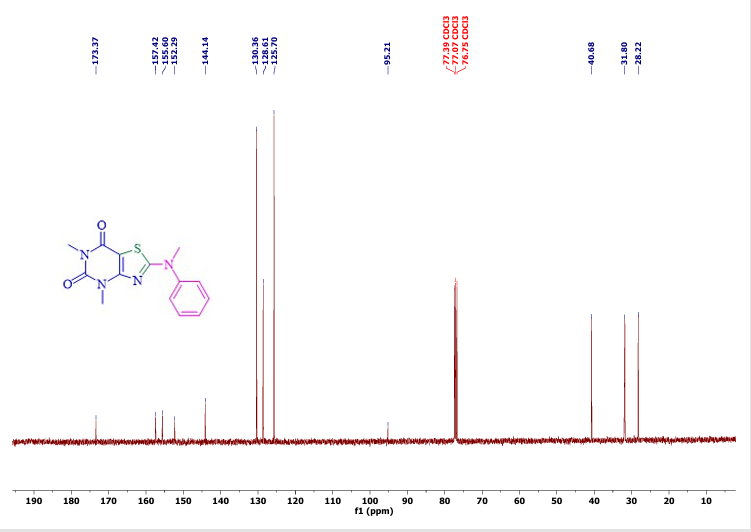


**^13^C-NMR of 4,6-dimethyl-2-(methyl(phenyl)amino)thiazolo[4,5-*d*]pyrimidine-5,7(4H,6H) -dione (Table 2, 4z).**


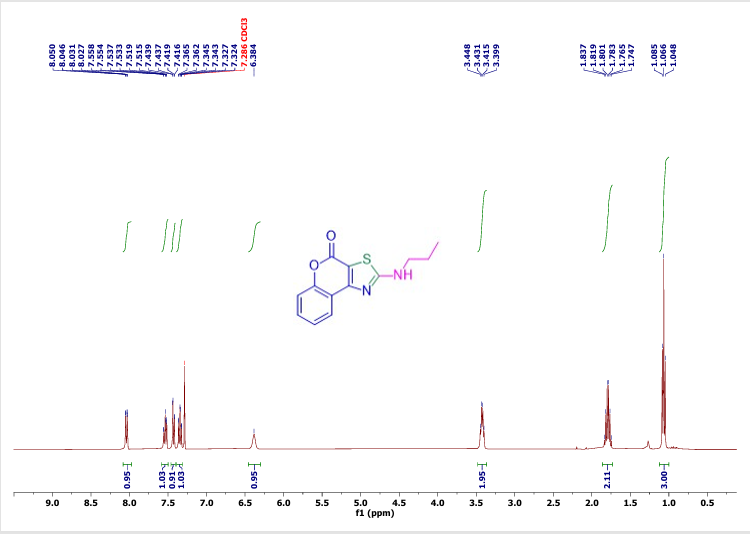


**^1^H-NMR of 2-(propylamino)-4H-chromeno[4,3-*d*]thiazol-4-one (Table 2, 4aa).**


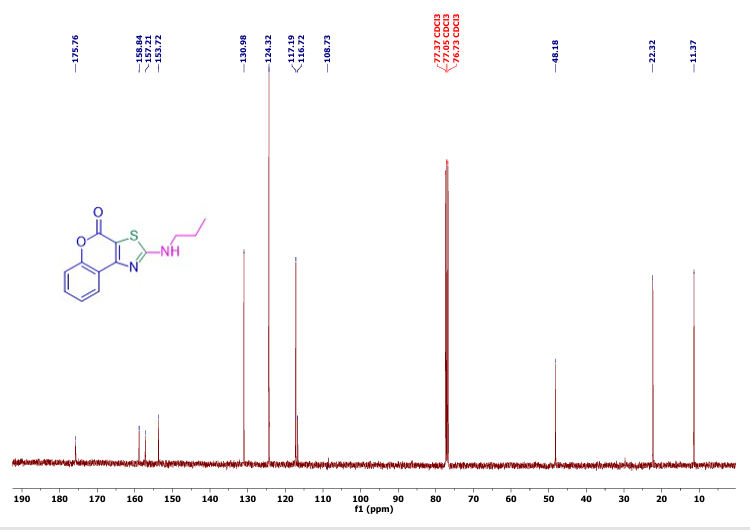


**^13^C-NMR of 2-(propylamino)-4H-chromeno[4,3-*d*]thiazol-4-one (Table 2, 4aa).**
